# Expression of complement and toll-like receptor pathway genes is associated with malaria severity in Mali: a pilot case control study

**DOI:** 10.1186/s12936-016-1189-6

**Published:** 2016-03-09

**Authors:** Rafal S. Sobota, Antoine Dara, Jessica E. Manning, Amadou Niangaly, Jason A. Bailey, Abdoulaye K. Kone, Mahamadou A. Thera, Abdoulaye A. Djimdé, Guy Vernet, Philippe Leissner, Scott M. Williams, Christopher V. Plowe, Ogobara K. Doumbo

**Affiliations:** Department of Molecular Physiology and Biophysics, Vanderbilt Genetics Institute, Vanderbilt University, Nashville, TN USA; Department of Genetics, Geisel School of Medicine, Dartmouth College, Hanover, NH USA; Department of Epidemiology of Parasitic Diseases, Malaria Research and Training Center, University of Sciences, Techniques and Technology of Bamako, Bamako, Mali; Division of Malaria Research, Institute for Global Health, University of Maryland School of Medicine, Baltimore, MD USA; Department of Medicine, Brigham and Women’s Hospital, Boston, MA USA; Pasteur Institute of Cameroon, Yaounde, Cameroon, Fondation Merieux, Lyon, France; BioMerieux, Grenoble, France

**Keywords:** Malaria, Genetic Epidemiology, Microarray, *Plasmodium*, Mali

## Abstract

**Background:**

The host response to infection by *Plasmodium falciparum*, the parasite most often responsible for severe malaria, ranges from asymptomatic parasitaemia to death. The clinical trajectory of malaria is influenced by host genetics and parasite load, but the factors determining why some infections produce uncomplicated malaria and some proceed to severe disease remain incompletely understood.

**Methods:**

To identify molecular markers of severe falciparum malaria, human gene expression patterns were compared between children aged 6 months to 5 years with severe and uncomplicated malaria who were enrolled in a case–control study in Bandiagara, Mali. Microarrays were used to obtain expression data on severe cases and uncomplicated controls at the time of acute disease presentation (five uncomplicated and five severe), 1 week after presentation (three uncomplicated and three severe) and treatment initiation, and in the subsequent dry season (late convalescence, four uncomplicated and four severe). This is a pilot study for the first use of microarray technology in Mali.

**Results:**

Complement and toll-like receptor (TLR) pathways were differentially expressed, with severe cases showing higher expression of the *C1q*, *TLR2*, *TLR4*, *TLR8*, and *CR1* genes. Other genes previously associated with malaria pathogenesis, *GZMB*, *FOS* and *HSPA6*, were also higher among severe cases. *TLR2*, *TLR4*, *TLR8*, *CR1*, *GZMB*, *FOS*, and *HSPA6* genes were expressed at lower levels in severe cases at late convalescence.

**Conclusions:**

Overexpression of genes previously associated with uncomplicated malaria was associated with severe disease. Low baseline expression of these genes may represent candidate markers for severe malaria. Despite the small sample size, results of this pilot study offer promising targets for follow-up analyses.

**Electronic supplementary material:**

The online version of this article (doi:10.1186/s12936-016-1189-6) contains supplementary material, which is available to authorized users.

## Background

Malaria continues to be a significant cause of morbidity and mortality with an estimated 438,000 deaths and 214 million cases in 2015 [1]. The response to infection by *Plasmodium falciparum*, the parasite responsible for most severe malaria, is varied and complex, ranging from asymptomatic parasitaemia to death. It has been estimated that out of 400 people who are bitten by *P. falciparum* infected *Anopheles* mosquitoes, 200 will be infected, 100 will develop the uncomplicated form of the disease, two will develop severe symptoms, and one will die [2]. Numerous factors affect the clinical manifestation of malaria, including a patient’s age [3], level of *P. falciparum* transmission [4], parasite virulence [5], and gene expression levels in both the host and the parasite [6, 7], among others [8]. Uncomplicated malaria is typically defined by fever accompanied by a blood smear positive for asexual *P. falciparum.* Severe malaria manifestations include severe anaemia, hypoglycaemia, acidosis, neurological symptoms from cerebral involvement, end-organ damage and/or respiratory distress [9]. The biological bases for differences in clinical manifestations remain largely unexplained.

Selective pressure of *P. falciparum* on human evolution has helped to shape the human genome; in addition, the *P. falciparum* and human genomes have co-evolved because sub-clinical infection or uncomplicated disease is more advantageous to the parasite than severe disease [10]. Examples of selection on the host genome include sickle cell trait (HbS), thalassaemias, haemoglobin C, and other erythrocyte variants [11]. These variants have been argued to play a more prominent role than environmental factors in the outcome of malaria infection [12]. Despite the importance attributed to genetically determined host responses, relatively few genetic variants have been identified that affect the clinical trajectory of infection. This is highlighted by the observation that sickle cell trait, the most prominent protective genetic mechanism, only explains 2 % of the total variation in response to *P. falciparum* infection [11]. To address this, analyses of human genomic variation using genome-wide level data have been performed to identify additional factors [13], but because of linkage disequilibrium patterns in Africans, such genome-wide analyses are problematic [14]. Alternative approaches that specifically target human genes in toto may be more fruitful. One technically feasible design is to assess genome-wide levels of expression to gauge the most important genes in response to infection.

Few studies to date have used microarray technology to identify host genes involved in response to *P. falciparum* infection. One study demonstrated a possible role for up-regulation of neutrophil and erythrocytic activity in 22 Kenyan children with fever and *P. falciparum* parasitaemia or other acute febrile illnesses [15]. In another study, toll-like receptors were shown to be induced by malarial infection irrespective of prior exposure, demonstrating the same pattern of expression in 22 experimentally infected, naïve volunteers in the USA and 22 naturally infected Cameroonians [16]. Although generally informative regarding innate and humoral immunity at a single time point, these studies provide data on temporally static measures against varied ethnic backgrounds, or only consider *P. falciparum* infection as a single clinical entity. Both of these factors may be critical in evaluating the results of expression studies.

To address the possible complicating factors of both the temporal differences in gene expression and host genetic diversity, a study in a high-transmission, ethnically homogeneous endemic region of Bandiagara, Mali was carried out. This provides a potentially powerful perspective from which to study host immune responses. By using a case–control study design, with cases of severe malaria compared to controls of uncomplicated malaria, the transcriptional profiles of these divergent host responses can be compared to detect differentially expressed gene clusters.

## Methods

### Study population

Children of both sexes aged between 6 months and 5 years, residing in Bandiagara, Mali and presenting to the Bandiagara District Hospital with acute clinical malaria were eligible for enrolment. Prior to enrolment, informed consent was obtained from parents or legal guardians. Other eligibility criteria included meeting definitions of either uncomplicated or severe falciparum malaria and availability for the entire six-month study period. World Health Organization (WHO) criteria for uncomplicated and severe malaria were used [9]. The presence of either respiratory distress, anaemia (<15 % haematocrit or <5 g/dL haemoglobin), hypoglycaemia (<40 mg/dL), persistent vomiting, or the inability to eat or drink necessitating parenteral feeding also qualified the patient as having severe malaria [9]. Exclusion criteria included the presence of any disease with clinical features similar to severe malaria (e.g., meningitis), a prior splenectomy or prolonged use of immunosuppressants, and any immunological intervention such as a vaccine or administration of immunoglobulins in the 6 months prior to enrolment in the study. Diagnoses were made during the August-December malaria transmission season.

### Sample collection

Blood was collected from each study participant (2.5 mL) in a PAXgeneTM Blood RNA tube (PreAnalytix, Germany) that stabilizes mRNA [17], and 20 µL was used to prepare a Giemsa- stained blood smear to confirm *P. falciparum* infection. The PAXgeneTM tubes were kept at room temperature overnight in accordance with the manufacturer’s instructions (>2 h), then kept at −20 °C for 24 h and finally transferred to −80 °C for long-term storage. RNA was extracted in accordance with protocol using the PAXgeneTM Blood RNA kit. The quality of the RNA samples was evaluated with the use of Agilent RNA 6000 nano kit (Agilent Technologies, Germany) and the corresponding Agilent 2100 Bioanalyzer, using the RNA integrity number (RIN) metric [18]. The A260/A280 ratio was also used to assess RNA purity. All microarray analyses were carried out at the Malaria Research and Training Centre in Bamako, Mali. The Affymetrix Human Genome U133 Plus 2.0 GeneChip platform and protocols (Santa Clara, USA) were used for the expression analysis. The Affymetrix GeneChip^®^ Scanner 3000 was used to analyse the chips and Affymetrix GeneChip Operating Software version 1.2 (GCOS) was used to manage Affymetrix GeneChip data.

### Microarray analysis

Data were analysed using R statistical software and the BioConductor ‘limma’ package [19]. Preprocessing and normalization were carried out using the robust multi-array average (RMA) [20]. Quality control measures were assessed by analysing GAPDH-6 and actin ratios as they compare to scaling factors, as well as evaluating the RNA degradation patterns for the various chips. Hierarchical clustering analyses were performed to check for any probe co-regulation patterns [19]. In this pilot study representing the first use of microarray technology in Mali, the use of Bonferroni or false discovery rate multiple-testing adjustments was not appropriate for analysing this small dataset (Additional file 1: Figure S1). Instead the data were analysed using the criteria of greater than two-fold change [log_2_(2) = 1] and a significance level of p < 0.05 for testing the null hypothesis of whether the two sets are the same. To assess whether any specific biological pathways were over- or under-expressed the probes matching the above criteria were filtered using the Kyoto Encyclopedia of Genes and Genomes [21] pathway filter in the statistical software R. The KEGG analyses were carried out for the probes matching these criteria separately during the three time points of the study: acute illness, convalescence and late convalescence. The probes matching the criteria for the acute illness time point were also filtered using Ingenuity Pathway Analysis (Ingenuity Systems, Redwood City, CA, USA), and their expression was followed over the remaining two time points. Only the probes whose functions could be defined by Ingenuity Pathway Analysis were included in this analysis.

### qRT-PCR analysis

Real-time PCR was carried out on samples from seven individuals (four severe cases, three uncomplicated controls) for whom RNA remained after the microarray analysis (microarray analysis was carried out again on only those seven individuals for this comparison). Ten genes were analysed, eight matching the significance threshold of greater than two-fold change [log_2_(2) = 1] and a p value of <0.05 and two were housekeeping genes (beta-actin and GAPDH). The selected genes were: CYP1B1, C1QB, CD163, CCR2, CLEC4D, NAIP, TLR2, and IL18R1. Pre-designed primer and probe sets for all genes were obtained from Applied Biosystems [22]. RT-PCR was carried out using the StepOnePlus™ Real-Time PCR System (Applied Biosystems), with TaqMan^**®**^ reagents in accordance with the manufacturer’s recommendations. TaqMan^**®**^ One-Step RT-PCR Master Mix Reagents Kit (PN 4304437) was used. Briefly, 50 ng of total RNA was used as a template for the one-step RT-PCR assay. The PCR conditions were as follows: 30 min at 48 °C, 15 s at 95 °C, 1 min at 60 °C, 40 cycles. Data were analysed using ABI software to generate delta Ct values, and the delta Ct method was used with GAPDH as Ref. [23]. For each gene, at least one uncomplicated and one severe malaria patient had to have expression data for all of the tests done in triplicate to be included in the final analysis. For genes that had multiple probes in the Gene Chip, expression values were averaged for comparison with PCR data. The RT-PCR was carried out at Genomics/Affymetrix unit of the Malaria Research and Training Centre in Bamako, Mali.

### Ethics approval

This study was approved by the ethics committee of the Faculty of Medicine, Pharmacy and Odonto-Stomatology, University of Sciences, Techniques and Technology of Bamako, Mali.

## Results

### Study population characteristics

Twenty-three uncomplicated malaria controls and 24 severe cases (with cerebral and other severe symptoms) were enrolled in this study. Five severe cases with cerebral symptoms and five uncomplicated malaria controls were chosen for pilot microarray studies of gene expression on the day of presentation at the clinic (acute illness time point). Three each of controls and cases returned seven days later to submit blood samples for a gene expression analysis a week later (convalescence time point). The clinical characteristics of the acute illness and convalescence time point patients in the study are summarized in Table 1 (Additional file 2: Table S1).

**Table 1:** Clinical characteristics of patients enrolled for the acute illness and convalescence time points

Patient	Gender	Age (years)	Symptoms during acute illness	Symptoms in convalescence
Severe malaria cases				
2	Male	3	Convulsions	None
4	Male	4	Obtundation	None
6	Female	3	Prostration, lethargy	None
9	Male	2	Coma, convulsions	None
12	Female	3.5	Prostration, lethargy	None
Uncomplicated malaria controls				
1	Female	5	Diarrhoea	Cough
3	Female	1.5	Cough	Cough
4	Male	4	Headache, chills, vomiting	–
7	Male	3	Chills	None
12	Female	3	Headache, abdominal pain, cough	Lethargy, abdominal pain

All patients were febrile and parasitaemic for *P. falciparum* at the acute time point and all were afebrile and aparasitaemic at the convalescent time point except uncomplicated control patient 4 (data not available)

Due to loss to follow-up and development of other disease between the relevant time points, eight different patients were followed to study the late convalescence, low transmission dry season gene expression following either severe cerebral (four patients) or uncomplicated (four patients) malarial disease. These patients were closely followed to assure that no other clinical episodes occurred between their acute illness time point presentation and their late convalescence follow-up. The clinical data for this group are summarized in Table 2 (Additional file 3: Table S2).

**Table 2:** Clinical characteristics of patients enrolled for the late convalescence time point

Patient	Gender	Age (years)	Symptoms during acute illness	Symptoms in late convalescence	Time since acute illness (days)
Severe malaria cases					
8	Female	2	Convulsions	None	165
10	Male	4	Prostration/lethargy	None	162
17	Female	3	Prostration/lethargy	None	147
18	Female	4	Obtundation	None	145
Uncomplicated malaria controls					
2	Male	3	Diarrhoea, vomiting	None	154
6	Male	3	Diarrhoea, vomiting	None	153
14	Male	2	Vomiting	None	137
23	Female	5	Headache	None	134

All patients were febrile and parasitaemic for *P. falciparum* during the acute illness (except severe malaria case 10, axillary T = 36.8 °C) and all patients were afebrile and aparasitaemic at late convalescence (except uncomplicated malaria control patient 2, axillary T = 37.7 °C)

### Top differentially expressed genes during acute illness

A total of 215 probes representing 134 genes were differentially expressed between severe and uncomplicated cases and matched the inclusion criteria of >twofold change and a p value <0.05. The complete list of these probes is included in Additional file 4: Table S3. Furthermore, 37 probes corresponding to 33 genes showed differential expression at a p value <0.005 and a greater than twofold change (Table 3), and four probes, representing *MB21D1, SAMSN1, PLBD1,* and *MKRN1* genes, had a p value <0.001. These top four genes have not been previously associated with malaria pathogenesis and/or severity. Of the remaining probes, four were in three genes, *HSPA6*, *TLR8* and *FOS*, previously identified in experiments for malaria severity in human or murine systems [24–26]. Two probes interrogated the expression of the *HSPA6* gene (p values of 0.0020 and 0.0040), with single association in *TLR8* and *FOS* (p values of 0.0038 and 0.0037, respectively) (Table 3).

**Table 3:** Significant genes differentiating between severe and uncomplicated malaria at the acute illness (function designated by Ingenuity Pathway Analysis)

Gene	Probe	Description	Acute illness log (fold change)	p value
MB21D1	1559051_s_at	Mab-21 domain containing 1	−1.482461	3.64E−04
SAMSN1	1555638_a_at	SAM domain, SH3 domain and nuclear localization signals 1	−1.265117	4.52E−04
PLBD1	218454_at	Phospholipase B domain containing 1	−1.016165	6.47E−04
MKRN1	209845_at	Makorin ring finger protein 1	1.078957	9.25E−04
PSMF1	201053_s_at	Proteasome (prosome, macropain) inhibitor subunit 1 (PI31)	1.088499	1.40E−03
HEMGN	223670_s_at	Haemogen	1.782966	1.41E−03
KREMEN1	227250_at	Kringle containing transmembrane protein 1	−1.004677	1.47E−03
FPR2	210772_at	Formyl peptide receptor 2	−1.121253	1.56E−03
BCL6	215990_s_at	B cell CLL/lymphoma 6	−1.244554	1.71E−03
HSPA6	117_at	Heat shock 70 kDa protein 6 (HSP70B’)	−1.358852	2.02E−03
CCR2	207794_at	Chemokine (C–C motif) receptor 2	−1.041326	2.11E−03
FPR2	210773_s_at	Formyl peptide receptor 2	−1.227297	2.23E−03
WNK1	1555068_at	WNK lysine deficient protein kinase 1	1.507248	2.25E−03
HLA-DPA1	211990_at	Major histocompatibility complex, class II, DP alpha 1	−1.370427	2.40E−03
SOS1	212777_at	Son of sevenless homologue 1 (*Drosophila*)	−1.564278	2.41E−03
SAMSN1	220330_s_at	SAM domain, SH3 domain and nuclear localization signals 1	−1.184775	2.47E−03
GNLY	205495_s_at	Granulysin	−1.246373	2.66E−03
TMEM165	226825_s_at	Transmembrane protein 165	−1.055969	2.72E−03
EIF5A	201123_s_at	Eukaryotic translation initiation factor 5A	2.023113	2.80E−03
CCR2	206978_at	Chemokine (C–C motif) receptor 2	−1.346736	2.94E−03
C14orf45	220173_at	Chromosome 14 open reading frame 45	1.49005	3.20E−03
ITGAM	205786_s_at	integrin, alpha M (complement component 3 receptor 3 sub-unit)	−1.038506	3.30E−03
BPGM	203502_at	2,3-bisphosphoglycerate mutase	1.360559	3.34E−03
FECH	203116_s_at	Ferrochelatase	1.547674	3.42E−03
IRAK3	213817_at	Interleukin-1 receptor-associated kinase 3	−1.445589	3.52E−03
DUSP1	201041_s_at	Dual specificity phosphatase 1	−1.168216	3.64E−03
FOS	209189_at	FBJ murine osteosarcoma viral oncogene homolog	−1.062789	3.69E−03
TLR8	220832_at	Toll-like receptor 8	−1.131984	3.80E−03
FOSL2	228188_at	FOS-like antigen 2	−1.020796	3.89E−03
SRRD	213608_s_at	SRR1 domain containing	1.110322	3.90E−03
NLRC4	1552553_a_at	NLR family, CARD domain containing 4	−1.001566	3.91E−03
HSPA6	213418_at	Heat shock 70 kDa protein 6 (HSP70B’)	−1.327448	3.98E−03
PSMF1	201052_s_at	Proteasome (prosome, macropain) inhibitor subunit 1 (PI31)	1.021332	4.11E−03
MS4A6A	219666_at	Membrane-spanning 4-domains, subfamily A, member 6A	−1.031532	4.29E−03
DUSP3	201537_s_at	Dual specificity phosphatase 3	−1.024139	4.36E−03
EGLN1	224314_s_at	Egl nine homologue 1 (*Caenorhabditis elegans*)	−1.036334	4.37E−03
STEAP4	1556185_a_at	STEAP family member 4	−1.385834	4.86E−03

### KEGG pathway analysis of the acute illness time point samples

**Fig. 1:**
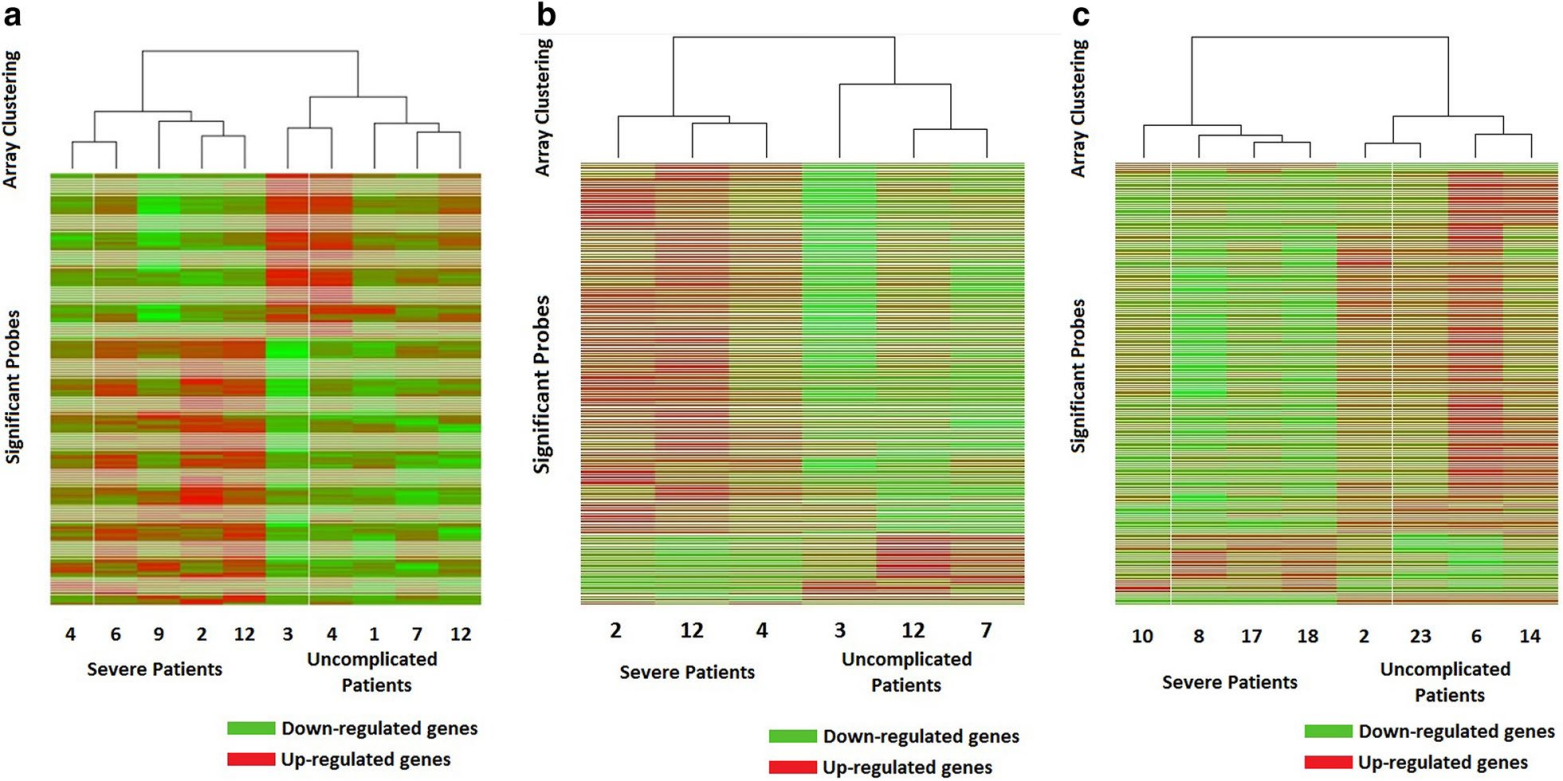
Hierarchical clustering of arrays using Pearson’s correlations on probes with a fold change greater than two and a p value <0.05. Down-regulated probes are in *green*, up-regulated probes are in *red*. **a** Heat map for the 215 probes matching the criteria for the acute illness patients; **b** Heat map for the 362 probes matching the criteria for the convalescence patients; **c** Heat map for the 340 probes matching the criteria for the dry season time point

A KEGG analysis based on the 215 significant probes showed that the malaria pathway had the second-highest odds ratio (Table 4). A hierarchical clustering heat map demonstrates the co-regulation of the 215 significant probes (Fig. 1a), and reveals the degree of separation between severe cases and uncomplicated ones. The odds ratios for the three top pathways, *Staphylococcus aureus* infection, malaria, and leishmaniasis were 15.2, 11.8 and 10.3, respectively, and all had p values of less than 0.001. Five of the 86 probed genes in the malaria pathway showed differential expression between the severe and uncomplicated case groups, with the expected value of 1. Both toll-like receptors two and four (TLR2, TLR4), part of the KEGG malaria pathway, had higher expression in the severe cases compared to the uncomplicated malaria group (2.07-fold higher for TLR2, 2.27- and 2.46-fold higher for the TLR4 probes). The other three significant genes associated with malaria in KEGG were the Complement Receptor 1 (2.05-fold higher expression in severe cases) along with glycophorin A and B (2.89- and 2.88-fold higher uncomplicated case expression, respectively). Since *Staphylococcus aureus* infection and leishmaniasis are infectious diseases, and each had a similar odds ratio to malaria, the top three pathways were assessed for any overlap in significant probes. The leishmaniasis pathway had three probes in common with malaria (TLR2, TLR4, CR1), while *Staphylococcus aureus* infection had one (CR1). Other significant KEGG pathways of relevance to malaria pathogenesis included complement and coagulation cascades and antigen processing and presentation with odds ratios of 6.4 and 4.5, respectively.

**Table 4:** Top KEGG pathways differentially expressing between severe cases and uncomplicated controls during acute illness

KEGG pathway	No. genes	Expected count^a^	Observed count^b^	Odds ratio	p value
*Staphylococcus aureus* infection	47	1	6	15.197	<0.001
Leishmaniasis	66	1	6	10.342	<0.001
Malaria	48	1	5	11.807	<0.001
Systemic lupus erythematosus	104	1	5	5.067	0.005
Allograft rejection	33	0	3	9.758	0.005
Complement and coagulation cascades	66	1	4	6.386	0.005
Graft-versus-host disease	35	0	3	9.145	0.006
Type I diabetes mellitus	39	0	3	8.122	0.008
Toxoplasmosis	121	1	5	4.308	0.009
Rheumatoid arthritis	79	1	4	5.264	0.01
Cytokine-cytokine receptor interaction	234	3	7	3.145	0.011
Hematopoietic cell lineage	83	1	4	4.993	0.011
Auto-immune thyroid disease	47	1	3	6.634	0.013
Chagas disease	98	1	4	4.183	0.02
Chemokine signaling pathway	165	2	5	3.094	0.03
Asthma	26	0	2	7.976	0.031
Antigen processing and presentation	67	1	3	4.541	0.034
Cell adhesion molecules (CAMs)	119	1	4	3.404	0.038
Renal cell carcinoma	70	1	3	4.335	0.038
MAPK signaling pathway	248	3	6	2.464	0.047

^a^ Expected count, the number of genes in the pathway that would be expected to be differentially expressed by chance

^b^ Observed count, the number of genes in the pathway observed to be differentially expressed

### KEGG pathway analyses of the convalescence and late convalescence time point samples

Analyses of differentially expressed KEGG pathways during convalescence revealed that the ‘malaria’ pathway was no longer significant at the <0.05 level when comparing uncomplicated *versus* severe patients (Additional file 5: TableS4). KEGG assessment of expression differences in the two groups at the late convalescence time point showed that the malaria pathway was again statistically significant (Additional file 6: Table S5). Hierarchical clustering heat maps were created for both the convalescence (Fig. 1b) and the late convalescence (Fig. 1c) time points, and the separation of severe from uncomplicated cases observed during acute illness was maintained. For the three severe and three uncomplicated case patients followed from acute disease to convalescence, the list of KEGG pathways differentiated at the 0.05 level includes the antigen processing and presentation pathway. During acute illness, this pathway had an odds ratio of 4.5 and a p value of 0.034, and it is still significant at convalescence seven days later with an odds ratio of 8.5 and a p value of 0.002 (Additional file 5: Table S4). With only one gene expected to show significance at the given threshold, HLA-DQA1 (3.97-fold higher expression in severe cases), HLA-DQB1 (10.34-fold higher expression in severe cases), HLA-DPA1 (2.01-fold higher expression in uncomplicated cases), and KLRC3 (3.39-fold higher expression in severe cases) all matched the above stated criteria. The unrelated cases and controls from the low transmission, late convalescence time point list malaria as the 12th most significantly differentiated pathway with an odds ratio of 4.1 and a p value of 0.043 (Additional file 6: Table S5). Three genes were observed to be expressed above the predefined threshold, CD36 (4.38- and 3.81-fold higher expression in the uncomplicated cases for two probes), IL18R (2.30-fold higher expression in uncomplicated cases) and TLR4 (2.94- and 2.61-fold higher expression in uncomplicated cases), where only one was expected.

### Ingenuity pathway analysis of the acute illness time point samples

**Fig. 2:**
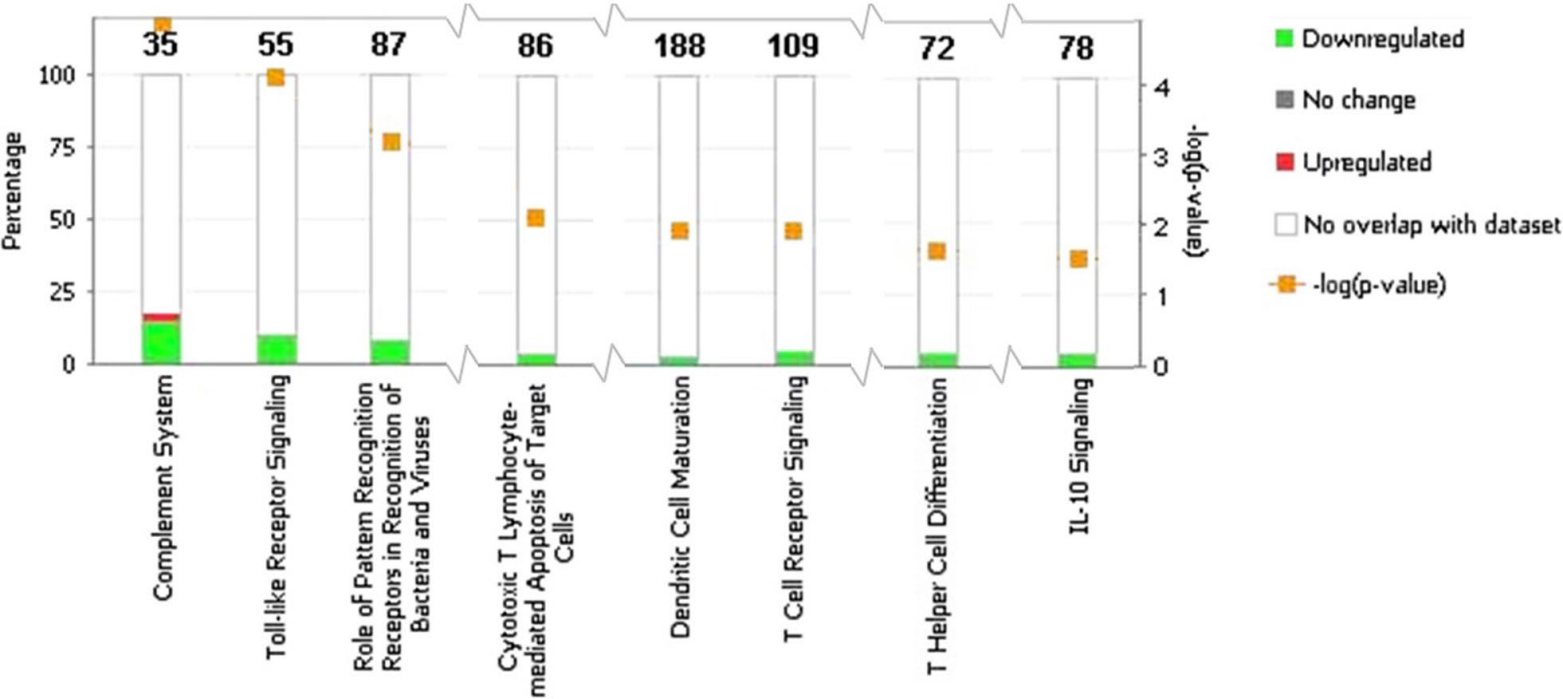
*Stacked bar chart* displaying the top canonical pathways found to be differentially represented in comparing gene expression in uncomplicated controls to severe cases during acute illness. Total number of genes in each pathway is displayed above each bar, in *bold*. Pathways are ranked by statistical significance. Only those considered pertinent to the malaria phenotype are displayed: Complement system (highest statistical significance), toll-like receptor signalling (2nd), role of pattern recognition receptors in recognition of bacteria and viruses (3rd), cytotoxic T lymphocyte-mediated apoptosis of target cells (11th), dendritic cell maturation (13th), T cell receptor signalling (14th), T helper cell differentiation (18th) and IL-10 signalling (20th) [27]

The top canonical pathways produced by applying Ingenuity Pathway Analysis to significant probes at acute illness were the complement system, toll-like receptor signaling and role of pattern recognition receptors in recognition of bacteria and viruses. Figure 2 shows a stacked bar chart of these pathways and other significant pathways considered to be relevant to the pathogenesis of malaria: Cytotoxic T lymphocyte-mediated apoptosis of target cells (11th ranked pathway in terms of significance), dendritic cell maturation (13th), T cell receptor signaling (14th), T helper cell differentiation (18th), and IL-10 signaling (20th) [27]. Of the significant probes that overlap with the above listed canonical pathways, the great majority shows lower expression in the patients who developed uncomplicated disease as compared to those who had severe symptoms. Differentially expressed probes form these canonical pathways that were previously shown to play a role in severe malaria pathogenesis were TLR2, TLR4, TLR8, HSPA6, CR1, C1qA, C1qB, C1qC, FOS, and GZMB. The TLR probes showed a higher expression in severe malaria cases during acute illness (2.07-fold for TLR2, 2.19-fold for TLR8, 2.27 and 2.46-fold for the TLR4 probes). HSPA6 levels were also higher in severe cases by 2.56-fold during this time point, as was CR1, which was expressed at a higher level in severe cases by 2.04. C1q is comprised of three chains, all of which were found to be expressed at higher levels in the severe patients (C1qA by 2.79-fold, C1qB by 3.12-fold and C1qC by 2.64-fold). The *FOS* and *GZMB* genes were also found to be expressed at elevated levels in severe malaria cases in this study (by 2.09- and 2.31-fold, respectively).

### Expression of genes involved in acute illness phenotype differentiation during convalescence and late convalescence

**Fig. 3:**
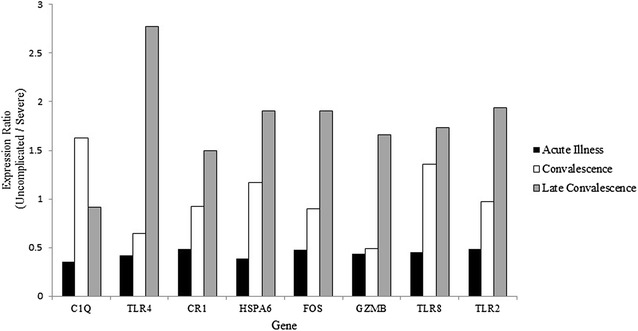
Longitudinal expression of molecular markers. Uncomplicated to severe expression ratios of eight severe malaria candidate markers at the acute illness time point, convalescence and late convalescence. Average expression values were used for the two significant TLR4 probes and separately C1Q sub-units A, B, and C

Longitudinal expression dynamics of the probes relevant to severe *versus* uncomplicated malaria presentation at acute illness from the section above were evaluated during convalescence. Eleven probes corresponding to eight genes were analysed; these genes were previously found to be experimentally relevant in severe malaria. Figure 3 displays the evolution of the expression of these genes over the three time points of this study. Seven of the eight genes showed characteristics that make them reasonable targets as molecular markers of severe malaria.

TLR probes showed a higher expression in severe malaria cases during acute illness (2.07-fold for TLR2, 2.19-fold for TLR8, 2.27 and 2.46-fold for TLR4 probes). By convalescence, most of the probes still showed higher expression in severe cases, but by a smaller margin (1.03-fold for TLR2, 1.85- and 1.33-fold for the TLR4 probes), with the exception of the TLR8 probe which showed a higher expression in the uncomplicated cases (1.36-fold). This trend continued into late convalescence, where all probes showed higher expression in uncomplicated cases as compared to severe (1.94-fold for TLR2, 1.73-fold for TLR8, 2.61- and 2.94-fold for the TLR4 probes). HSPA6 levels were higher in severe cases by 2.56-fold during the acute illness time point, by convalescence uncomplicated cases were 1.17-fold higher and in late convalescence uncomplicated cases were expressed 1.90 times higher. During acute illness, CR1 was expressed at a higher level in severe cases by 2.04, 7 days later during convalescence it was 1.09 times higher is severe cases, and by late convalescence CR1 was expressed at higher levels in the uncomplicated case patients by 1.50-fold. FOS expression was higher in severe cases during acute illness as well as during convalescence, by 2.08 and 1.11, respectively, but by late convalescence, the uncomplicated cases that showed a 1.90-fold higher expression. GZMB had a 2.33-fold higher expression in severe cases during acute illness, 2.04-fold higher expression in severe cases during convalescence, but by the late convalescence time point uncomplicated cases expressed the gene 1.66-fold higher.

C1q was the only protein out of the eight candidates that did not follow a similar expression profile. Three probes corresponding to the chains of the C1q protein show a change from having higher expression in the severe patients during acute illness (C1qA by 2.79-fold, C1qB by 3.12-fold, C1qC by 2.64-fold) to showing higher expression in uncomplicated cases during convalescence (C1qA by 1.65-fold, C1qB by 1.54-fold, C1qC by 1.69-fold). By the late convalescence time point, the difference in these probes between uncomplicated and severe cases was seemingly indistinguishable between the groups (C1qA 1.10-fold higher expression in severe, C1qB no difference, C1qC 1.20-fold higher expression in severe).

### qRT-PCR validation

The Spearman Rho for the correlation of the GeneChip and RT-PCR expression was 0.87. The Pearson’s correlation between the GeneChip expression data and RT-PCR values was 0.72, but there were not enough observations to assure a normal distribution (Additional file 7: Table S6). The correlations indicate consistency between the two approaches considering the low fold change inclusion criteria for the expression data.

## Discussion

Seven molecular marker candidate genes of severe malaria were identified in this study. Evaluating the baseline expression of these genes may identify individuals at risk prior to infection. When comparing gene expression between uncomplicated and severe malaria patients at acute illness, the list of differentially expressed genes was enriched for those consistently associated with malaria in prior studies. Previous studies reported differences in expression of toll-like receptors and the complement system in healthy as compared to uncomplicated *P. falciparum* malaria [7, 27, 28]. This study demonstrated that TLR and complement systems are not just implicated in human gene expression in response to uncomplicated symptoms of a *P. falciparum* infection, but also appear to play a role in the pathophysiology of severe cerebral malaria. These genes were expressed at significantly higher levels in patients presenting with severe symptoms during acute illness. All of the sub-units of *C1q* were elevated in severe cases, as was *CR1, TLR2, TLR4*, and *TLR8*. These results are consistent with prior studies showing dysregulation of innate immunity and inflammatory mechanisms in severe malaria morbidity [23, 25, 27, 28].

The relevance of both complement and TLR pathways is well established in response to malaria. The classical pathway of complement activation occurs via binding of antibodies in immune complexes initiated by C1q [29]. CR1 has been shown to be protective against *P. falciparum* infections in low concentrations, but when over-expressed it can lead to rosetting of blood cells and subsequent severe malaria [8, 28, 29]. Single nucleotide polymorphisms (SNPs) in *CR1* also associate with disease manifestation in India [28]. The elevation of TLR probes in severe patients in this study also recapitulates previous findings [29]. TLR4 has been associated with clinical malaria outcomes in human studies as its suppression in dendritic cells has been associated with uncomplicated *P. falciparum* infection in Malian children of the Dogon ethnicity [30]. TLR8 is thought to prime and potentiate the inflammatory response to pathogens, thereby contributing to the pathogenesis of severe malaria [25]. *HSPA6* expression has also been previously implicated in malaria pathogenesis, although it has never been considered when studying the severe versus uncomplicated phenotype. Upon *P. falciparum* infection of nucleated erythroid precursor cells, the orthochromatic cells, the level of HSPA6 was shown to be significantly increased above what it was in controls [24].

This study also replicates in human *P. falciparum* malaria findings from mouse models for severe *versus* uncomplicated *Plasmodium berghei* malaria, which implicated genes involved in both complement and TLR, along with *FOS* and *GZMB* [26]. In a murine model, a *TLR 2/4*^−*/*−^ genotype leads to increased resistance against developing experimentally induced *P. berghei* cerebral malaria [31]. The c-fos protein was found to be expressed at elevated levels in experimental murine *P. berghei*-induced cerebral malaria [26]. While the c-fos protein was also found to be slightly elevated in non-cerebral murine malaria cases, its level and extent of neurological involvement was far greater in the cerebral cases. This is the first study directly linking *FOS* expression levels to severe malaria in humans. Another mouse model found the *GZMB*^*−*/−^ genotype to be protective of *P. berghei* cerebral malaria [32].

The use of three time points throughout the evolution of disease permitted study of the changes in expression as disease progressed from the acute illness episode, through convalescence and into the late convalescence, low transmission season. Although the patient population is not the same for the acute and late convalescence time points, the results from the late convalescence time point can stand alone, as these are children who experienced severe *versus* uncomplicated malaria in the previous rainy season; therefore, differences in gene expression from this time point have a prognostic value. While the various C1Q probes appeared to have similar expression patterns, the TLR probes, GZMB, FOS, HSPA6, and CR1 were all expressed at higher levels in the uncomplicated cases during the late convalescence, dry season time point. The higher expression of the TLR genes, GZMB, FOS, HSPA6, and CR1, in uncomplicated malaria cases during the late convalescence time point can be interpreted two ways. It is possible that prolonged suppression of these genes following severe infection is causal. Considering that the average time elapsed since the acute illness presentation was 150 days, and no intermittent malaria episodes occurred, this scenario is unlikely; although it cannot be ruled out. Obtaining baseline expression levels, prior to the development of malaria, would have been more informative, however there are substantial logistical obstacles on account of the relatively low prevalence of cerebral malaria. It can also be argued that the late convalescence levels represent the baseline expression values for these individuals. Under this hypothesis, the baseline expression levels of these genes are candidate markers for developing severe malarial disease upon infection.

The WHO definition of severe malaria allows for a broad spectrum of clinical symptoms, ranging from respiratory distress to cerebral complications, amongst others [9]. While this is a clinically convenient method of clustering individuals and rationing appropriate care, it is problematic from the perspective of gene expression profiling, in that each clinical scenario of severe malaria spectrum may have a different underlying gene expression profile. All patients defined as severe cases at all time points of this study were classified based on cerebral complications; however, there is some heterogeneity even within this group (Tables 1, 2). Considering the broad spectrum of clinical features that fall into the severe malaria category, having all of the patients fall into the cerebral complications phenotype is a strength of this study.

In an experiment looking strictly into host gene expression, the differences in *P. falciparum* genetics and their interaction with host factors are a possible source of error [7]. The importance of parasite genetics in determining the clinical trajectory of disease has been demonstrated in previous studies delineating the effects of *var* gene expression in governing clinical outcomes [33–35]. Larger studies are needed to examine parasite genetic polymorphism and gene expression differences in relation to human gene expression and clinical malaria syndromes. Host circadian rhythm expression differences could have also added to the variation between the severe and uncomplicated cases of malaria as exact blood collection times were not measured, therefore, cases and controls could not be matched on this basis. It is also possible that the findings are confounded by variable white cell counts in the patient population; however, the capacity to separate the cells was not yet available in Mali during this pilot study. Furthermore, since the objective was to find easily tested molecular markers, whole blood analyses were implemented.

The availability and increasing affordability of genome-wide expression arrays has intensified the search of human molecular markers for susceptibility to severe malaria. The low incidence of severe malaria limits the potential for genome-wide approaches from a sequencing/genome-wide association perspective because of the sample size necessary to attain significance in such studies. The presence of various sub-types of severe malaria further complicates such studies due to phenotyping considerations, again lowering the potential sample size for a well-designed study. The uncomplicated malaria phenotype has been shown to be problematic in genome-wide association studies (GWAS), where *HBS* did not attain genome-wide significance in the initial screen [13]. Expression approaches such as the one described here might provide a better solution to unravelling severe malaria pathogenesis. Expression studies are more powered for finding relevant genes because they only carry out 22,000 independent tests, as opposed to the >1 million in GWAS, and gene expression is closer to the clinical phenotype than sequence variation. This is supported by the results of this study, as they were highly enriched for genes consistently associated with the phenotype in prior experiments, despite the small sample size. The results verify expression findings previously limited to the mouse model organism, or human data only previously associated with uncomplicated disease. The gene clusters highlighted by this study can guide future inquiries into unravelling the intricacies of the protective host immune response against severe malarial infection. Understanding the genetics of the host response, via gene expression, may accelerate the identification of clinically useful prognostic indicators, and ultimately, inform treatment and prevention guidelines for severe malaria.

## Conclusions

This study suggests that overexpression of genes previously associated with uncomplicated malaria may also be associated with severe disease. Here, models of severe malaria were replicated in humans for the first time, and genes previously thought only to contribute to uncomplicated malaria were implicated in severe disease. In particular, genes in the complement and toll-like receptor pathways were expressed at higher levels in severe cases during acute illness. The longitudinal component of this study demonstrated that 7 days after presentation and treatment, the levels of these complement and toll-like receptor genes were indistinguishable in the uncomplicated and severe malaria patients. However, when expression of these genes was evaluated during the dry season following acute illness, patients with previous severe malaria showed lower levels of expression in these genes. These low baseline values should be evaluated as possible candidate biomarkers for severe malaria.

## Additional files

10.1186/s12936-016-1189-6 Power as a function of sample size for an Affymetrix Human Genome U133 Plus 2.0 GeneChip with alpha 0.05, FDR 0.05, and a standard deviation of 0.6 under the assumption that 90% of the genes on the chip remain undifferentiated in their expression.
10.1186/s12936-016-1189-6 Clinical characteristics of enrolled patients for the acute illness and convalescence time points.
10.1186/s12936-016-1189-6 Clinical characteristics of enrolled patients for the Dry Season time point.
10.1186/s12936-016-1189-6 All statistically significant probes for testing gene expression difference between severe cases and uncomplicated controls during acute illness (function designated by Ingenuity Pathway Analysis).
10.1186/s12936-016-1189-6 Top differentially expressed KEGG pathways between severe cases and uncomplicated controls during convalescence.
10.1186/s12936-016-1189-6 Top differentially expressed KEGG pathways between severe cases and uncomplicated controls during late convalescence.
10.1186/s12936-016-1189-6 RT-PCR validation of GeneChip expression data with RT-PCR (probes for CYP1B1, IL18R1 and C1QB failed to consistently amplify, failing the inclusion criteria; therefore they were not included in the final validation analysis).

## References

[1] WHO (2015). World Malaria Report.

[2] Marsh K (1992). Malaria–a neglected disease?. Parasitology.

[3] Djimdé AA, Doumbo OK, Traore O, Guindo AB, Kayentao K, Diourte Y (2003). Clearance of drug-resistant parasites as a model for protective immunity in *Plasmodium falciparum* malaria. Am J Trop Med Hyg.

[4] Snow RW, Omumbo JA, Lowe B, Molyneux CS, Obiero JO, Palmer A (1997). Relation between severe malaria morbidity in children and level of *Plasmodium falciparum* transmission in Africa. Lancet.

[5] Gupta S, Hill AV, Kwiatkowski D, Greenwood AM, Greenwood BM, Day KP (1994). Parasite virulence and disease patterns in *Plasmodium falciparum* malaria. Proc Natl Acad Sci USA.

[6] McGuire W, Hill AV, Allsopp CE, Greenwood BM, Kwiatkowski D (1994). Variation in the TNF-alpha promoter region associated with susceptibility to cerebral malaria. Nature.

[7] Idaghdour Y, Quinlan J, Goulet JP, Berghout J, Gbeha E, Bruat V (2012). Evidence for additive and interaction effects of host genotype and infection in malaria. Proc Natl Acad Sci USA.

[8] Miller LH, Baruch DI, Marsh K, Doumbo OK (2002). The pathogenic basis of malaria. Nature.

[9] World Health Organization (2000). Communicable diseases cluster. severe falciparum malaria. Trans R Soc Trop Med Hyg.

[10] Hill AV, Jepson A, Plebanski M, Gilbert SC (1997). Genetic analysis of host-parasite coevolution in human malaria. Philos Trans R Soc Lond B Biol Sci.

[11] Kwiatkowski DP (2005). How malaria has affected the human genome and what human genetics can teach us about malaria. Am J Hum Genet.

[12] Ntoumi F, Kwiatkowski DP, Diakite M, Mutabingwa TK, Duffy PE (2007). New interventions for malaria: mining the human and parasite genomes. Am J Trop Med Hyg.

[13] Jallow M, Teo YY, Small KS, Rockett KA, Deloukas P, Clark TG (2009). Genome-wide and fine-resolution association analysis of malaria in West Africa. Nat Genet.

[14] Teo YY, Small KS, Kwiatkowski DP (2010). Methodological challenges of genome-wide association analysis in Africa. Nat Rev Genet.

[15] Griffiths MJ, Shafi MJ, Popper SJ, Hemingway CA, Kortok MM, Wathen A (2005). Genomewide analysis of the host response to malaria in Kenyan children. J Infect Dis.

[16] Ockenhouse CF, Hu WC, Kester KE, Cummings JF, Stewart A, Heppner DG (2006). Common and divergent immune response signaling pathways discovered in peripheral blood mononuclear cell gene expression patterns in presymptomatic and clinically apparent malaria. Infect Immun.

[17] Rainen L, Oelmueller U, Jurgensen S, Wyrich R, Ballas C, Schram J (2002). Stabilization of mRNA expression in whole blood samples. Clin Chem.

[18] Schroeder A, Mueller O, Stocker S, Salowsky R, Leiber M, Gassmann M (2006). The RIN: an RNA integrity number for assigning integrity values to RNA measurements. BMC Mol Biol.

[19] Tuimala J. DNA Microarray data analysis using Bioconductor. CSC—IT Center for Science Ltd 2008.

[20] Smyth GK. Linear models and empirical bayes methods for assessing differential expression in microarray experiments. Stat Appl Genet Mol Biol. 2004, 3:Article3. 10.2202/1544-6115.1027 16646809

[21] Genomes KEoGa. http://www.genome.jp/kegg/. Accessed 19 Mar 2012.

[22] Biosystems A. https://bioinfo.appliedbiosystems.com/genome-database/. Accessed 12 Nov 2012.

[23] Livak KJ, Schmittgen TD (2001). Analysis of relative gene expression data using real-time quantitative PCR and the 2(-Delta Delta C(T)) method. Methods.

[24] Tamez PA, Liu H, Wickrema A, Haldar KP (2011). *falciparum* modulates erythroblast cell gene expression in signaling and erythrocyte production pathways. PLoS ONE.

[25] Franklin BS, Ishizaka ST, Lamphier M, Gusovsky F, Hansen H, Rose J (2011). Therapeutical targeting of nucleic acid-sensing Toll-like receptors prevents experimental cerebral malaria. Proc Natl Acad Sci USA.

[26] Ma N, Harding AJ, Pamphlett R, Chaudhri G, Hunt NH (1997). Increased c-fos expression in the brain during experimental murine cerebral malaria: possible association with neurologic complications. J Infect Dis.

[27] Perkins DJ, Were T, Davenport GC, Kempaiah P, Hittner JB, Ong’echa JM (2011). Severe malarial anemia: innate immunity and pathogenesis. Int J Biol Sci.

[28] Sinha S, Jha GN, Anand P, Qidwai T, Pati SS, Mohanty S (2009). CR1 levels and gene polymorphisms exhibit differential association with falciparum malaria in regions of varying disease endemicity. Hum Immunol.

[29] Silver KL, Higgins SJ, McDonald CR, Kain KC (2010). Complement driven innate immune response to malaria: fuelling severe malarial diseases. Cell Microbiol.

[30] Arama C, Giusti P, Bostrom S, Dara V, Traore B, Dolo A (2011). Interethnic differences in antigen-presenting cell activation and TLR responses in Malian children during *Plasmodium falciparum* malaria. PLoS ONE.

[31] Kordes M, Matuschewski K, Hafalla JC (2011). Caspase-1 activation of interleukin-1beta (IL-1beta) and IL-18 is dispensable for induction of experimental cerebral malaria. Infect Immun.

[32] Haque A, Best SE, Unosson K, Amante FH, de Labastida F, Anstey NM (2011). Granzyme B expression by CD8 + T cells is required for the development of experimental cerebral malaria. J Immunol.

[33] Kaestli M, Cockburn IA, Cortes A, Baea K, Rowe JA, Beck HP (2006). Virulence of malaria is associated with differential expression of *Plasmodium falciparum var* gene subgroups in a case-control study. J Infect Dis.

[34] Rottmann M, Lavstsen T, Mugasa JP, Kaestli M, Jensen AT, Muller D (2006). Differential expression of *var* gene groups is associated with morbidity caused by *Plasmodium falciparum* infection in Tanzanian children. Infect Immun.

[35] Kyriacou HM, Stone GN, Challis RJ, Raza A, Lyke KE, Thera MA (2006). Differential *var* gene transcription in *Plasmodium falciparum* isolates from patients with cerebral malaria compared to hyperparasitaemia. Mol Biochem Parasitol.

