# Supplementary material for: Expression of complement and toll-like receptor pathway genes is associated with malaria severity in Mali: a pilot case control study
Source: Malar J. 2016 Mar 9;15:150. doi: 10.1186/s12936-016-1189-6 (PMC4784286; doi:10.1186/s12936-016-1189-6)
Supplement: Supplementary file 1 — 10.1186/s12936-016-1189-6 Power as a function of sample size for an Affymetrix Human Genome U133 Plus 2.0 GeneChip with alpha 0.05, FDR 0.05, and a standard deviation of 0.6 under the assumption that 90% of the genes on the chip remain undifferentiated in their expression. [file 12936_2016_1189_MOESM1_ESM.docx]

**Supplemental Figure**

**
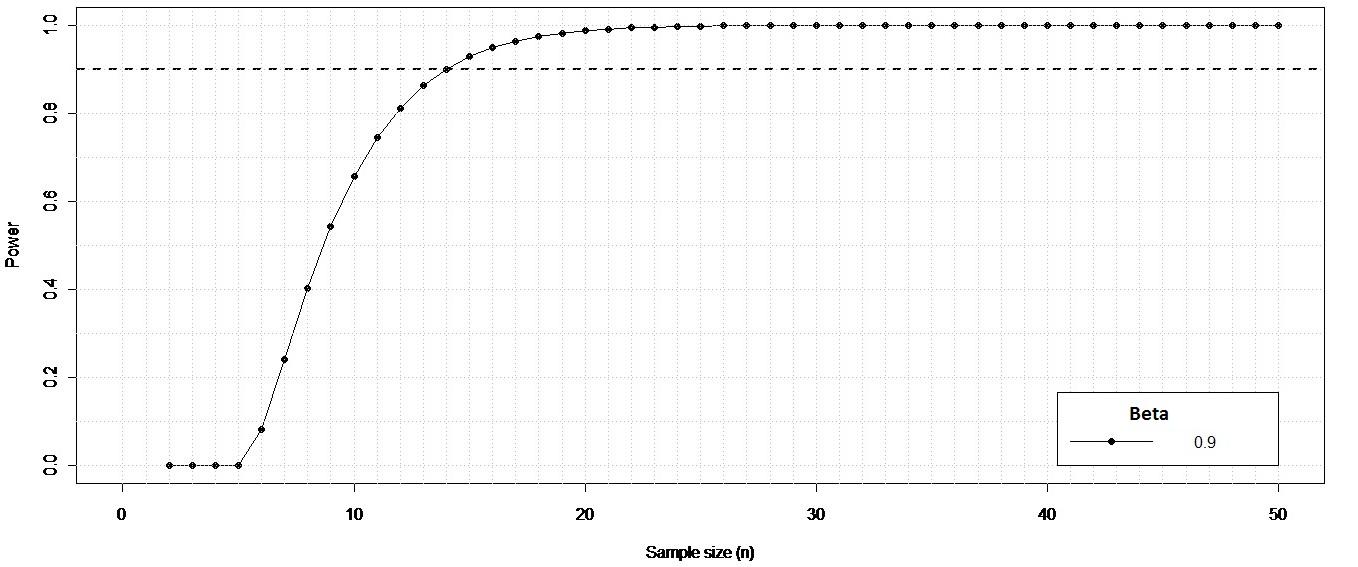
**

**Figure S1.** Power as a function of sample size for an Affymetrix Human Genome U133 Plus 2.0 GeneChip with alpha 0.05, FDR 0.05, and a standard deviation of 0.6 under the assumption that 90% of the genes on the chip remain undifferentiated in their expression.^18^ A priori power calculations require 14 cases and controls to be analyzed with a chip that carries 54,675 probes in order to attain power of 0.9, an alpha value of 0.05 along with a 0.05 false discovery rate assuming a 0.6 standard deviation and two-fold differences in expression. Therefore barring any large scale expression differences between the cases and controls, a sample size of 5 cases and 5 controls will not produce data significant enough to stay under the 0.05 p-value threshold following the Benjamini and Hotchberg adjustment. The power for this sample size is 0.0011, therefore as expected none of the data points remained significant after adjusting for FDR, which is why a two-fold expression difference and <0.05 p value criteria were used in this study.
