# Supplementary material for: Expression of complement and toll-like receptor pathway genes is associated with malaria severity in Mali: a pilot case control study
Source: Malar J. 2016 Mar 9;15:150. doi: 10.1186/s12936-016-1189-6 (PMC4784286; doi:10.1186/s12936-016-1189-6)
Supplement: Supplementary file 2 — 10.1186/s12936-016-1189-6 Clinical characteristics of enrolled patients for the acute illness and convalescence time points. [file 12936_2016_1189_MOESM2_ESM.docx]

**Table S1: Clinical characteristics of enrolled patients for the acute illness and convalescence time points**

| Patient | Status | Symptoms on day 0 | Sex | Age | Temp on day 0 | Med. 2 weeks prior to visit | Treatment prescribed on day 0 | Temperature at day 7 | Parasitemia on day 7 | Symptoms on day 7 | Parasitemia day 0 (parasites/ul) | Hemoglobin day0 (g/dL) |
| --- | --- | --- | --- | --- | --- | --- | --- | --- | --- | --- | --- | --- |
| 2 | Severe | Convulsions | M | 3 | 39 | N | Quinine, Diazepam | 36.6 | N | N | 5100 | 8.6 |
| 4 | Severe | Obtundation | M | 4 | 38.6 | Y | Quinine, Glucose | 36.7 | N | N | 95375 | 8.4 |
| 6 | Severe | Prostation/Lethargy | F | 3 | 39.6 | N | Quinine, Glucose | NA | N | N | 17325 | 6.9 |
| 9 | Severe | Coma/Convulsions | M | 2 | 39.6 | N | Quinine, Glucose | NA | N | N | 1850 | 7.2 |
| 12 | Severe | Prostation/Lethargy | F | 3.5 | 38 | N | Quinine, Glucose | 37.1 | N | N | 175 | 9.8 |
| 1 | Simple | Diarrhea | F | 5 | 36.9 | N | ACT, Paracetamol | 36.6 | N | Cough, bilat. rhonchi | 32925 | 14.4 |
| 3 | Simple | Cough | F | 1.5 | 39.5 | Y | ACT, Paracetamol | 36.5 | N | Cough | 21825 | 6.7 |
| 4 | Simple | Headache, Chills, Vomiting | M | 4 | 38.4 | N | ACT, Paracetamol, Iron | NA | NA | NA | 1625 | 6.2 |
| 7 | Simple | Chills | M | 3 | 39.4 | N | ACT, Paracetamol | 37.3 | N | N | 103200 | 9.2 |
| 12 | Simple | Headache, Abd Pain, Cough | F | 3 | 39.4 | N | ACT, Paracetamol | 36.9 | N | Lethargy, stomach pain | 210000 | 7.3 |
