# Supplementary material for: Expression of complement and toll-like receptor pathway genes is associated with malaria severity in Mali: a pilot case control study
Source: Malar J. 2016 Mar 9;15:150. doi: 10.1186/s12936-016-1189-6 (PMC4784286; doi:10.1186/s12936-016-1189-6)
Supplement: Supplementary file 3 — 10.1186/s12936-016-1189-6 Clinical characteristics of enrolled patients for the Dry Season time point. [file 12936_2016_1189_MOESM3_ESM.docx]

**Table S2: Clinical characteristics of enrolled patients for the Dry Season time point**

| Patient | Status | Symptoms on day 0 | Sex | Age | Temp on day 0 | Parasitemia day (parasites/ul) | Med. 2 weeks prior to visit | Treatment prescribed on day 0 | Temperature during dry season | Parasitemia during dry season | Symptoms during dry season | Days since clinical dx | Parasites/ul Dry season | Dry Hemoglobin (g/dL) |
| --- | --- | --- | --- | --- | --- | --- | --- | --- | --- | --- | --- | --- | --- | --- |
| 8 | Severe | Convulsions | F | 2 | 39.6 | 48775 | Paracetamol | Quinine, Diazepam | 36.6 | N | None | 165 | 0 | 8.9 |
| 10 | Severe | Prostation/Lethargy | M | 4 | 36.8 | 29650 | Paracetamol | Quinine, Diazepam | 36.5 | N | None | 162 | 0 | 12.6 |
| 17 | Severe | Prostation/Lethargy | F | 3 | 39.6 | 38325 | Paracetamol | Artesiane, Aspegic | 36.8 | N | None | 147 | 3075 | 10.9 |
| 18 | Severe | Obtundation | F | 4 | 39.9 | 39625 | Paracetamol | Quinine, Diazepam | 36.1 | N | None | 145 | 0 | 9.4 |
| 2 | Simple | Diarrhea, Vomiting | M | 3 | 37.5 | 72450 | Paracetamol | ACT, Paracetamol | 37.7 | N | None | 154 | 7575 | 9.8 |
| 6 | Simple | Diarrhea, Vomiting | M | 3 | 38.2 | 165000 | Paracetamol | Quinine, ACT, Paracet. | 35.9 | N | None | 153 | 0 | 11.3 |
| 14 | Simple | Vomiting | M | 2 | 38.2 | 96750 | N | ACT, Aspegic, Paracet. | 36.7 | N | None | 137 | 0 | 12.0 |
| 23 | Simple | Headache | F | 5 | 37.6 | 600 | Paracetamol | ACT, Paracetamol | 37.2 | N | None | 134 | 0 | 11.2 |

**Table S3 All statistically significant probes for testing gene expression difference between severe cases and uncomplicated controls during acute illness (function designated by Ingenuity Pathway Analysis)**

| **Probe ID** | **Gene** | **Description** | **log(fold change)** | **p value** |
| --- | --- | --- | --- | --- |
| 117_at | HSPA6 | heat shock 70kDa protein 6 (HSP70B') | -1.35885 | 0.002017 |
| 1552553_a_at | NLRC4 | NLR family, CARD domain containing 4 | -1.00157 | 0.003906 |
| 1552583_s_at | ABCC13 | ATP-binding cassette, sub-family C (CFTR/MRP), member 13, pseudogene | 1.128954 | 0.036698 |
| 1552670_a_at | PPP1R3B | protein phosphatase 1, regulatory subunit 3B | -1.11426 | 0.007891 |
| 1552772_at | CLEC4D | C-type lectin domain family 4, member D | -1.57352 | 0.018025 |
| 1552773_at | CLEC4D | C-type lectin domain family 4, member D | -1.51267 | 0.03204 |
| 1553723_at | GPR97 | G protein-coupled receptor 97 | -1.00118 | 0.041318 |
| 1553861_at | TCP11L2 | t-complex 11 (mouse)-like 2 | 1.137328 | 0.009487 |
| 1554508_at | PIK3AP1 | phosphoinositide-3-kinase adaptor protein 1 | -1.05857 | 0.014124 |
| 1555068_at | WNK1 | WNK lysine deficient protein kinase 1 | 1.507248 | 0.002245 |
| 1555638_a_at | SAMSN1 | SAM domain, SH3 domain and nuclear localization signals 1 | -1.26512 | 0.000452 |
| 1556185_a_at | STEAP4 | STEAP family member 4 | -1.38583 | 0.004856 |
| 1559051_s_at | MB21D1 | Mab-21 domain containing 1 | -1.48246 | 0.000364 |
| 1559573_at | LOC100506229 | hypothetical LOC100506229 | -2.02288 | 0.03258 |
| 1560679_at | LOC100506328 | hypothetical LOC100506328 | -1.56791 | 0.013894 |
| 1568780_at | LOC497257 | hypothetical LOC497257 | -1.05313 | 0.017759 |
| 201041_s_at | DUSP1 | dual specificity phosphatase 1 | -1.16822 | 0.003644 |
| 201052_s_at | PSMF1 | proteasome (prosome, macropain) inhibitor subunit 1 (PI31) | 1.021332 | 0.004112 |
| 201053_s_at | PSMF1 | proteasome (prosome, macropain) inhibitor subunit 1 (PI31) | 1.088499 | 0.001398 |
| 201122_x_at | EIF5A | eukaryotic translation initiation factor 5A | 1.045381 | 0.007668 |
| 201123_s_at | EIF5A | eukaryotic translation initiation factor 5A | 2.023113 | 0.002804 |
| 201249_at | SLC2A1 | solute carrier family 2 (facilitated glucose transporter), member 1 | 1.226607 | 0.027045 |
| 201250_s_at | SLC2A1 | solute carrier family 2 (facilitated glucose transporter), member 1 | 1.381069 | 0.040169 |
| 201537_s_at | DUSP3 | dual specificity phosphatase 3 | -1.02414 | 0.004364 |
| 201940_at | CPD | carboxypeptidase D | -1.12948 | 0.022789 |
| 201942_s_at | CPD | carboxypeptidase D | -1.33144 | 0.007384 |
| 201943_s_at | CPD | carboxypeptidase D | -1.10191 | 0.036253 |
| 202430_s_at | PLSCR1 | phospholipid scramblase 1 | -1.03577 | 0.012683 |
| 202436_s_at | CYP1B1 | cytochrome P450, family 1, subfamily B, polypeptide 1 | -1.23657 | 0.018207 |
| 202437_s_at | CYP1B1 | cytochrome P450, family 1, subfamily B, polypeptide 1 | -1.66659 | 0.005696 |
| 202441_at | ERLIN1 | ER lipid raft associated 1 | -1.05239 | 0.041369 |
| 202444_s_at | ERLIN1 | ER lipid raft associated 1 | -1.06249 | 0.019336 |
| 202708_s_at | HIST2H2BE | histone cluster 2, H2be | -1.05482 | 0.048115 |
| 202922_at | GCLC | glutamate-cysteine ligase, catalytic subunit | 1.287958 | 0.007823 |
| 202953_at | C1QB | complement component 1, q subcomponent, B chain | -1.64092 | 0.031419 |
| 202974_at | MPP1 | membrane protein, palmitoylated 1, 55kDa | 1.095314 | 0.036845 |
| 203116_s_at | FECH | ferrochelatase | 1.547674 | 0.003416 |
| 203502_at | BPGM | 2,3-bisphosphoglycerate mutase | 1.360559 | 0.003343 |
| 203645_s_at | CD163 | CD163 molecule | -2.22533 | 0.008908 |
| 203973_s_at | CEBPD | CCAAT/enhancer binding protein (C/EBP), delta | -1.0217 | 0.01836 |
| 204187_at | GMPR | guanosine monophosphate reductase | 1.143445 | 0.046489 |
| 204560_at | FKBP5 | FK506 binding protein 5 | -1.06493 | 0.019455 |
| 204720_s_at | DNAJC6 | DnaJ (Hsp40) homolog, subfamily C, member 6 | 1.592844 | 0.025488 |
| 204860_s_at | NAIP | NLR family, apoptosis inhibitory protein | -1.45983 | 0.020844 |
| 204861_s_at | NAIP | NLR family, apoptosis inhibitory protein | -1.11548 | 0.021331 |
| 204924_at | TLR2 | toll-like receptor 2 | -1.05081 | 0.015466 |
| 205006_s_at | NMT2 | N-myristoyltransferase 2 | 1.037399 | 0.027581 |
| 205389_s_at | ANK1 | ankyrin 1, erythrocytic | 1.280485 | 0.049183 |
| 205390_s_at | ANK1 | ankyrin 1, erythrocytic | 1.554131 | 0.012932 |
| 205495_s_at | GNLY | granulysin | -1.24637 | 0.002665 |
| 205592_at | SLC4A1 | solute carrier family 4, anion exchanger, member 1 (erythrocyte membrane protein band 3, Diego blood group) | 1.258913 | 0.026053 |
| 205786_s_at | ITGAM (includes EG:16409) | integrin, alpha M (complement component 3 receptor 3 subunit) | -1.03851 | 0.003301 |
| 205900_at | KRT1 | keratin 1 | 1.436683 | 0.020963 |
| 205950_s_at | CA1 | carbonic anhydrase I | 1.725623 | 0.007122 |
| 206464_at | BMX | BMX non-receptor tyrosine kinase | -1.13424 | 0.045286 |
| 206522_at | MGAM | maltase-glucoamylase (alpha-glucosidase) | -1.01163 | 0.024929 |
| 206618_at | IL18R1 | interleukin 18 receptor 1 | -1.64143 | 0.014989 |
| 206698_at | XK | X-linked Kx blood group (McLeod syndrome) | 1.014307 | 0.011568 |
| 206974_at | CXCR6 | chemokine (C-X-C motif) receptor 6 | -1.01897 | 0.014958 |
| 206978_at | CCR2 | chemokine (C-C motif) receptor 2 | -1.34674 | 0.002939 |
| 206991_s_at | CCR5 | chemokine (C-C motif) receptor 5 | -1.0788 | 0.014547 |
| 207008_at | CXCR2 | chemokine (C-X-C motif) receptor 2 | -1.09498 | 0.009814 |
| 207275_s_at | ACSL1 | acyl-CoA synthetase long-chain family member 1 | -1.33457 | 0.017836 |
| 207338_s_at | ZNF200 | zinc finger protein 200 | -1.02999 | 0.029458 |
| 207387_s_at | GK | glycerol kinase | -1.27151 | 0.02265 |
| 207459_x_at | GYPB | glycophorin B (MNS blood group) | 1.494415 | 0.024318 |
| 207791_s_at | RAB1A | RAB1A, member RAS oncogene family | -1.00301 | 0.005529 |
| 207794_at | CCR2 | chemokine (C-C motif) receptor 2 | -1.04133 | 0.002106 |
| 208352_x_at | ANK1 | ankyrin 1, erythrocytic | 1.023101 | 0.029092 |
| 208451_s_at | C4B | complement component 4B (Chido blood group) | 1.765881 | 0.01218 |
| 208488_s_at | CR1 | complement component (3b/4b) receptor 1 (Knops blood group) | -1.03887 | 0.026963 |
| 209189_at | FOS | FBJ murine osteosarcoma viral oncogene homolog | -1.06279 | 0.003695 |
| 209273_s_at | ISCA1 | iron-sulfur cluster assembly 1 homolog (S. cerevisiae) | 1.094893 | 0.045562 |
| 209392_at | ENPP2 | ectonucleotide pyrophosphatase/phosphodiesterase 2 | -1.1497 | 0.015576 |
| 209480_at | HLA-DQB1 | major histocompatibility complex, class II, DQ beta 1 | -2.78201 | 0.03157 |
| 209735_at | ABCG2 | ATP-binding cassette, sub-family G (WHITE), member 2 | 1.419663 | 0.03259 |
| 209845_at | MKRN1 | makorin ring finger protein 1 | 1.078957 | 0.000925 |
| 209960_at | HGF | hepatocyte growth factor (hepapoietin A; scatter factor) | -1.39847 | 0.006502 |
| 210112_at | HPS1 (includes EG:114638) | Hermansky-Pudlak syndrome 1 | 1.146288 | 0.018369 |
| 210119_at | KCNJ15 | potassium inwardly-rectifying channel, subfamily J, member 15 | -1.13616 | 0.033481 |
| 210151_s_at | DYRK3 | dual-specificity tyrosine-(Y)-phosphorylation regulated kinase 3 | 1.025378 | 0.049406 |
| 210164_at | GZMB | granzyme B (granzyme 2, cytotoxic T-lymphocyte-associated serine esterase 1) | -1.20657 | 0.005892 |
| 210504_at | KLF1 | Kruppel-like factor 1 (erythroid) | 1.687688 | 0.017852 |
| 210746_s_at | EPB42 | erythrocyte membrane protein band 4.2 | 1.500856 | 0.017652 |
| 210772_at | FPR2 | formyl peptide receptor 2 | -1.12125 | 0.001556 |
| 210773_s_at | FPR2 | formyl peptide receptor 2 | -1.2273 | 0.002234 |
| 211372_s_at | IL1R2 | interleukin 1 receptor, type II | -2.09981 | 0.032008 |
| 211571_s_at | VCAN | versican | -1.15477 | 0.01001 |
| 211574_s_at | CD46 | CD46 molecule, complement regulatory protein | -1.13302 | 0.047014 |
| 211820_x_at | GYPA | glycophorin A (MNS blood group) | 1.303591 | 0.048908 |
| 211821_x_at | GYPA | glycophorin A (MNS blood group) | 1.532413 | 0.035832 |
| 211990_at | HLA-DPA1 | major histocompatibility complex, class II, DP alpha 1 | -1.37043 | 0.002396 |
| 212148_at | PBX1 | pre-B-cell leukemia homeobox 1 | 1.528451 | 0.030925 |
| 212192_at | KCTD12 | potassium channel tetramerisation domain containing 12 | -1.10503 | 0.01036 |
| 212602_at | WDFY3 | WD repeat and FYVE domain containing 3 | -1.0177 | 0.025199 |
| 212777_at | SOS1 | son of sevenless homolog 1 (Drosophila) | -1.56428 | 0.002406 |
| 212820_at | DMXL2 | Dmx-like 2 | -1.10462 | 0.021551 |
| 213418_at | HSPA6 | heat shock 70kDa protein 6 (HSP70B') | -1.32745 | 0.003983 |
| 213608_s_at | SRRD | SRR1 domain containing | 1.110322 | 0.003904 |
| 213724_s_at | PDK2 | pyruvate dehydrogenase kinase, isozyme 2 | 1.235411 | 0.025701 |
| 213757_at | EIF5A | eukaryotic translation initiation factor 5A | -1.13203 | 0.04263 |
| 213817_at | IRAK3 | interleukin-1 receptor-associated kinase 3 | -1.44559 | 0.003521 |
| 213934_s_at | ZNF23 | zinc finger protein 23 (KOX 16) | 1.235993 | 0.022353 |
| 214407_x_at | GYPB | glycophorin B (MNS blood group) | 1.6232 | 0.025513 |
| 214428_x_at | C4B | complement component 4B (Chido blood group) | 1.663075 | 0.009039 |
| 214433_s_at | SELENBP1 | selenium binding protein 1 | 1.292835 | 0.024764 |
| 214472_at | HIST1H3A | histone cluster 1, H3a | -1.35661 | 0.01691 |
| 214511_x_at | FCGR1B | Fc fragment of IgG, high affinity Ib, receptor (CD64) | -1.1051 | 0.038902 |
| 214590_s_at | UBE2D1 | ubiquitin-conjugating enzyme E2D 1 | -1.11855 | 0.023272 |
| 214706_at | ZNF200 | zinc finger protein 200 | -1.0834 | 0.007464 |
| 215049_x_at | CD163 | CD163 molecule | -2.30284 | 0.00677 |
| 215242_at | PIGC | phosphatidylinositol glycan anchor biosynthesis, class C | 1.38401 | 0.025327 |
| 215646_s_at | VCAN | versican | -1.16842 | 0.006501 |
| 215761_at | DMXL2 | Dmx-like 2 | -1.00802 | 0.020508 |
| 215990_s_at | BCL6 | B-cell CLL/lymphoma 6 | -1.24455 | 0.001712 |
| 216233_at | CD163 | CD163 molecule | -1.18121 | 0.017939 |
| 216317_x_at | RHCE/RHD | Rh blood group, D antigen | 1.155257 | 0.02433 |
| 216833_x_at | GYPB | glycophorin B (MNS blood group) | 1.529166 | 0.024352 |
| 216899_s_at | SKAP2 | src kinase associated phosphoprotein 2 | -1.02655 | 0.024062 |
| 217104_at | ST20 | suppressor of tumorigenicity 20 | -1.22745 | 0.03595 |
| 217437_s_at | TACC1 | transforming, acidic coiled-coil containing protein 1 | -1.04633 | 0.043888 |
| 218116_at | C9orf78 | chromosome 9 open reading frame 78 | 1.131912 | 0.009344 |
| 218141_at | UBE2O | ubiquitin-conjugating enzyme E2O | 1.092201 | 0.018805 |
| 218232_at | C1QA | complement component 1, q subcomponent, A chain | -1.48377 | 0.015729 |
| 218454_at | PLBD1 | phospholipase B domain containing 1 | -1.01617 | 0.000647 |
| 218864_at | TNS1 | tensin 1 | 1.40982 | 0.021017 |
| 218918_at | MAN1C1 | mannosidase, alpha, class 1C, member 1 | 1.022559 | 0.026804 |
| 219386_s_at | SLAMF8 | SLAM family member 8 | -1.03625 | 0.014004 |
| 219546_at | BMP2K | BMP2 inducible kinase | 1.033265 | 0.021759 |
| 219666_at | MS4A6A | membrane-spanning 4-domains, subfamily A, member 6A | -1.03153 | 0.004294 |
| 219672_at | AHSP | alpha hemoglobin stabilizing protein | 1.133232 | 0.018751 |
| 219890_at | CLEC5A | C-type lectin domain family 5, member A | -1.41646 | 0.032089 |
| 219938_s_at | PSTPIP2 | proline-serine-threonine phosphatase interacting protein 2 | -1.26104 | 0.010536 |
| 219975_x_at | OLAH | oleoyl-ACP hydrolase | -1.4701 | 0.044801 |
| 220034_at | IRAK3 | interleukin-1 receptor-associated kinase 3 | -1.06465 | 0.013564 |
| 220173_at | C14orf45 | chromosome 14 open reading frame 45 | 1.49005 | 0.003199 |
| 220330_s_at | SAMSN1 | SAM domain, SH3 domain and nuclear localization signals 1 | -1.18478 | 0.002472 |
| 220751_s_at | C5orf4 | chromosome 5 open reading frame 4 | 1.534989 | 0.006816 |
| 220832_at | TLR8 | toll-like receptor 8 | -1.13198 | 0.003801 |
| 221237_s_at | OSBP2 | oxysterol binding protein 2 | 1.210608 | 0.041056 |
| 221478_at | BNIP3L | BCL2/adenovirus E1B 19kDa interacting protein 3-like | 1.068535 | 0.007646 |
| 221627_at | TRIM10 | tripartite motif containing 10 | 1.230021 | 0.011252 |
| 221747_at | TNS1 | tensin 1 | 1.495251 | 0.027967 |
| 221748_s_at | TNS1 | tensin 1 | 1.586389 | 0.023444 |
| 221824_s_at | MARCH8 | membrane-associated ring finger (C3HC4) 8 | 1.026101 | 0.013806 |
| 221932_s_at | GLRX5 | glutaredoxin 5 | 1.016878 | 0.028989 |
| 222496_s_at | RBM47 | RNA binding motif protein 47 | -1.02925 | 0.026022 |
| 222687_s_at | ACER3 | alkaline ceramidase 3 | -1.02925 | 0.012769 |
| 222693_at | FNDC3B | fibronectin type III domain containing 3B | -1.02964 | 0.006019 |
| 222721_at | CNIH4 | cornichon homolog 4 (Drosophila) | -1.08283 | 0.01642 |
| 222895_s_at | BCL11B | B-cell CLL/lymphoma 11B (zinc finger protein) | 1.347469 | 0.037271 |
| 222945_x_at | OLAH | oleoyl-ACP hydrolase | -1.15684 | 0.015626 |
| 223124_s_at | PITHD1 | PITH (C-terminal proteasome-interacting domain of thioredoxin-like) domain containing 1 | 1.433289 | 0.008575 |
| 223204_at | FAM198B | family with sequence similarity 198, member B | -1.04469 | 0.013971 |
| 223432_at | OSBP2 | oxysterol binding protein 2 | 1.648538 | 0.008885 |
| 223597_at | ITLN1 | intelectin 1 (galactofuranose binding) | 1.5827 | 0.041377 |
| 223669_at | HEMGN | hemogen | 1.628387 | 0.034946 |
| 223670_s_at | HEMGN | hemogen | 1.782966 | 0.00141 |
| 223952_x_at | DHRS9 | dehydrogenase/reductase (SDR family) member 9 | -1.03108 | 0.016567 |
| 224009_x_at | DHRS9 | dehydrogenase/reductase (SDR family) member 9 | -1.12083 | 0.009459 |
| 224314_s_at | EGLN1 | egl nine homolog 1 (C. elegans) | -1.03633 | 0.004375 |
| 224341_x_at | TLR4 | toll-like receptor 4 | -1.18434 | 0.018445 |
| 224891_at | FOXO3 | forkhead box O3 | 1.034798 | 0.023177 |
| 224898_at | WDR26 | WD repeat domain 26 | 1.181848 | 0.045529 |
| 224905_at | WDR26 | WD repeat domain 26 | 1.187785 | 0.017186 |
| 225051_at | EPB41 | erythrocyte membrane protein band 4.1 (elliptocytosis 1, RH-linked) | 1.062133 | 0.005264 |
| 225056_at | SIPA1L2 | signal-induced proliferation-associated 1 like 2 | -1.40641 | 0.015878 |
| 225353_s_at | C1QC | complement component 1, q subcomponent, C chain | -1.39915 | 0.020331 |
| 225387_at | TSPAN5 | tetraspanin 5 | 1.275052 | 0.00898 |
| 225878_at | KIF1B | kinesin family member 1B | -1.03193 | 0.006232 |
| 226416_at | ERI1 (includes EG:361159) | exoribonuclease 1 | -1.16874 | 0.025507 |
| 226825_s_at | TMEM165 | transmembrane protein 165 | -1.05597 | 0.002719 |
| 227250_at | KREMEN1 | kringle containing transmembrane protein 1 | -1.00468 | 0.001465 |
| 227309_at | YOD1 | YOD1 OTU deubiquinating enzyme 1 homolog (S. cerevisiae) | 1.04528 | 0.040356 |
| 227769_at | GPR27 | G protein-coupled receptor 27 | -1.32022 | 0.017697 |
| 228188_at | FOSL2 | FOS-like antigen 2 | -1.0208 | 0.003894 |
| 228220_at | FCHO2 | FCH domain only 2 | -1.18086 | 0.036266 |
| 228758_at | BCL6 | B-cell CLL/lymphoma 6 | -1.03138 | 0.040476 |
| 228770_at | GPR146 | G protein-coupled receptor 146 | 1.196172 | 0.019806 |
| 228996_at | RC3H1 | ring finger and CCCH-type domains 1 | -1.02059 | 0.048389 |
| 229005_at | MCTP2 | multiple C2 domains, transmembrane 2 | -1.12717 | 0.011844 |
| 229228_at | CREB5 | cAMP responsive element binding protein 5 | -1.16456 | 0.020361 |
| 231874_at | FAM126B | family with sequence similarity 126, member B | -1.05708 | 0.032456 |
| 231933_at | MARCH8 | membrane-associated ring finger (C3HC4) 8 | 1.066229 | 0.017985 |
| 231982_at | C19orf77 | chromosome 19 open reading frame 77 | 1.085092 | 0.007797 |
| 231997_at | TBCEL | tubulin folding cofactor E-like | 1.006768 | 0.02002 |
| 232068_s_at | TLR4 | toll-like receptor 4 | -1.30127 | 0.022209 |
| 233126_s_at | OLAH | oleoyl-ACP hydrolase | -1.31599 | 0.029539 |
| 233587_s_at | SIPA1L2 | signal-induced proliferation-associated 1 like 2 | -1.34934 | 0.026226 |
| 234362_s_at | CTLA4 | cytotoxic T-lymphocyte-associated protein 4 | -1.24035 | 0.041415 |
| 235514_at | ASPRV1 | aspartic peptidase, retroviral-like 1 | -1.1891 | 0.032015 |
| 236081_at | SNCA | synuclein, alpha (non A4 component of amyloid precursor) | 1.440437 | 0.044911 |
| 238439_at | ANKRD22 | ankyrin repeat domain 22 | -1.32664 | 0.046381 |
| 238513_at | PRRG4 | proline rich Gla (G-carboxyglutamic acid) 4 (transmembrane) | -1.39075 | 0.048646 |
| 238858_at | TIFA | TRAF-interacting protein with forkhead-associated domain | -1.10957 | 0.019324 |
| 239206_at | CR1L | complement component (3b/4b) receptor 1-like | 1.631136 | 0.009521 |
| 241817_at | C3orf62 | chromosome 3 open reading frame 62 | -1.03078 | 0.019329 |
| 241881_at | OR2W3 | olfactory receptor, family 2, subfamily W, member 3 | 1.507999 | 0.047705 |
| 37145_at | GNLY | granulysin | -1.0927 | 0.00544 |
| 39729_at | PRDX2 | peroxiredoxin 2 | 1.046544 | 0.01548 |
| 59644_at | BMP2K | BMP2 inducible kinase | 1.08296 | 0.039044 |

**Table S4 Top differentially expressed KEGG pathways between severe cases and uncomplicated controls during convalescence**

| **Kegg Pathway** | **Size** | **Expected Count** | **Observed Count** | **Odds Ratio** | **p value** |
| --- | --- | --- | --- | --- | --- |
| Staphylococcus aureus infection | 47 | 0 | 5 | 16.401 | <0.001 |
| Asthma | 26 | 0 | 4 | 24.436 | <0.001 |
| Intestinal immune network for IgA production | 41 | 0 | 4 | 14.483 | <0.001 |
| Phagosome | 137 | 1 | 6 | 6.378 | 0.001 |
| Antigen processing and presentation | 67 | 1 | 4 | 8.459 | 0.002 |
| Allograft rejection | 33 | 0 | 3 | 13.044 | 0.002 |
| Graft-versus-host disease | 35 | 0 | 3 | 12.224 | 0.003 |
| Type I diabetes mellitus | 39 | 0 | 3 | 10.856 | 0.004 |
| Autoimmune thyroid disease | 47 | 0 | 3 | 8.867 | 0.006 |
| Chemokine signaling pathway | 165 | 1 | 5 | 4.197 | 0.01 |
| Cytokine-cytokine receptor interaction | 234 | 2 | 6 | 3.587 | 0.011 |
| Mismatch repair | 22 | 0 | 2 | 12.719 | 0.014 |
| Leishmaniasis | 66 | 1 | 3 | 6.168 | 0.016 |
| Viral myocarditis | 66 | 1 | 3 | 6.168 | 0.016 |
| Toxoplasmosis | 121 | 1 | 4 | 4.502 | 0.016 |
| Rheumatoid arthritis | 79 | 1 | 3 | 5.099 | 0.026 |

**Table S5 Top differentially expressed KEGG pathways between severe cases and uncomplicated controls during late convalescence**

| **KEGG Pathway** | **Size** | **Expected Count** | **Observed Count** | **Odds Ratio** | **p value** |
| --- | --- | --- | --- | --- | --- |
| Hematopoietic cell lineage | 83 | 1 | 7 | 5.982 | <0.001 |
| Toll-like receptor signaling pathway | 97 | 2 | 7 | 5.036 | 0.001 |
| Hepatitis C | 125 | 2 | 7 | 3.817 | 0.004 |
| RIG-I-like receptor signaling pathway | 66 | 1 | 5 | 5.194 | 0.004 |
| Cytosolic DNA-sensing pathway | 48 | 1 | 4 | 5.704 | 0.007 |
| NOD-like receptor signaling pathway | 52 | 1 | 4 | 5.224 | 0.01 |
| Cytokine-cytokine receptor interaction | 234 | 4 | 9 | 2.587 | 0.013 |
| African trypanosomiasis | 31 | 1 | 3 | 6.656 | 0.014 |
| PPAR signaling pathway | 64 | 1 | 4 | 4.168 | 0.02 |
| Type I diabetes mellitus | 39 | 1 | 3 | 5.168 | 0.025 |
| Nicotinate and nicotinamide metabolism | 17 | 0 | 2 | 8.196 | 0.031 |
| Malaria | 48 | 1 | 3 | 4.126 | 0.043 |
| Amyotrophic lateral sclerosis (ALS) | 50 | 1 | 3 | 3.949 | 0.048 |
| Apoptosis | 84 | 1 | 4 | 3.113 | 0.048 |

**Table S6 RT-PCR validation of GeneChip expression data with RT-PCR (probes for CYP1B1, IL18R1 and C1QB failed to consistently amplify, failing the inclusion criteria; therefore they were not included in the final validation analysis)**

| **Gene** | **Gene Chip Expression** | **RT-PCR Expression** |
| --- | --- | --- |
| CD163 | 0.38 | 0.73 |
| NAIP | 0.32 | 0.49 |
| TLR2 | 0.63 | 0.79 |
| CLEC4D | 0.38 | 0.52 |
| CCR2 | 0.34 | 0.34 |

**Supplemental Figure**

**
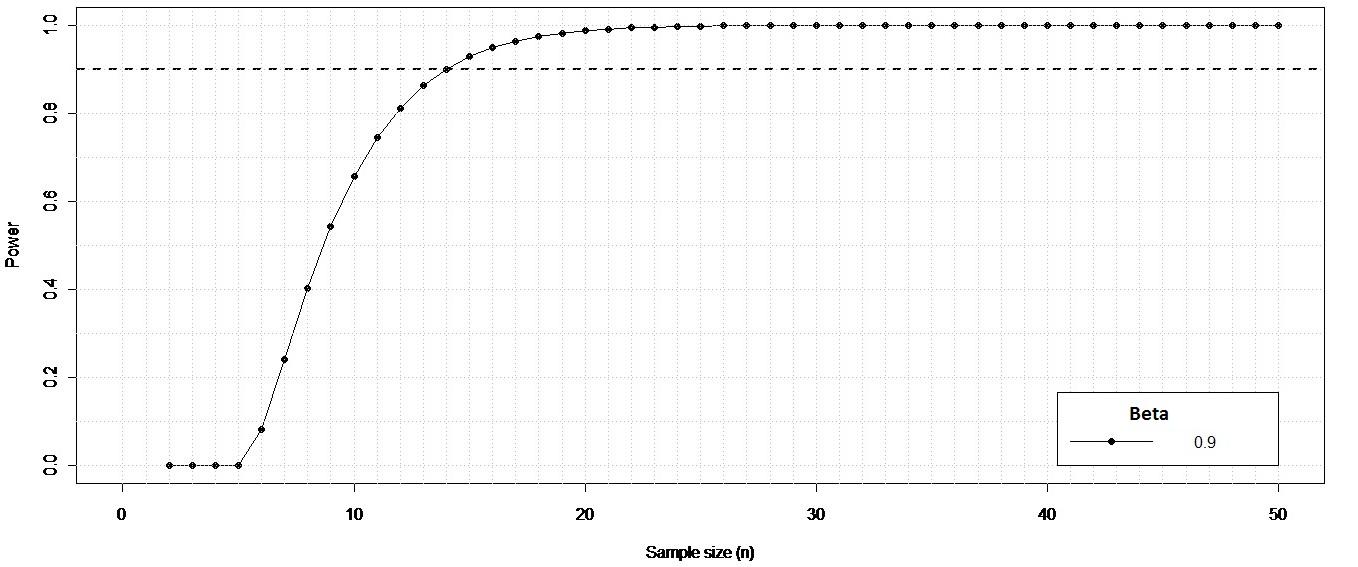
**

**Figure S1.** Power as a function of sample size for an Affymetrix Human Genome U133 Plus 2.0 GeneChip with alpha 0.05, FDR 0.05, and a standard deviation of 0.6 under the assumption that 90% of the genes on the chip remain undifferentiated in their expression.^18^ A priori power calculations require 14 cases and controls to be analyzed with a chip that carries 54,675 probes in order to attain power of 0.9, an alpha value of 0.05 along with a 0.05 false discovery rate assuming a 0.6 standard deviation and two-fold differences in expression. Therefore barring any large scale expression differences between the cases and controls, a sample size of 5 cases and 5 controls will not produce data significant enough to stay under the 0.05 p-value threshold following the Benjamini and Hotchberg adjustment. The power for this sample size is 0.0011, therefore as expected none of the data points remained significant after adjusting for FDR, which is why a two-fold expression difference and <0.05 p value criteria were used in this study.
