# Supplementary material for: Expression of complement and toll-like receptor pathway genes is associated with malaria severity in Mali: a pilot case control study
Source: Malar J. 2016 Mar 9;15:150. doi: 10.1186/s12936-016-1189-6 (PMC4784286; doi:10.1186/s12936-016-1189-6)
Supplement: Supplementary file 4 — 10.1186/s12936-016-1189-6 All statistically significant probes for testing gene expression difference between severe cases and uncomplicated controls during acute illness (function designated by Ingenuity Pathway Analysis). [file 12936_2016_1189_MOESM4_ESM.docx]

**Table S3 All statistically significant probes for testing gene expression difference between severe cases and uncomplicated controls during acute illness (function designated by Ingenuity Pathway Analysis)**

| **Probe ID** | **Gene** | **Description** | **log(fold change)** | **p value** |
| --- | --- | --- | --- | --- |
| 117_at | HSPA6 | heat shock 70kDa protein 6 (HSP70B') | -1.35885 | 0.002017 |
| 1552553_a_at | NLRC4 | NLR family, CARD domain containing 4 | -1.00157 | 0.003906 |
| 1552583_s_at | ABCC13 | ATP-binding cassette, sub-family C (CFTR/MRP), member 13, pseudogene | 1.128954 | 0.036698 |
| 1552670_a_at | PPP1R3B | protein phosphatase 1, regulatory subunit 3B | -1.11426 | 0.007891 |
| 1552772_at | CLEC4D | C-type lectin domain family 4, member D | -1.57352 | 0.018025 |
| 1552773_at | CLEC4D | C-type lectin domain family 4, member D | -1.51267 | 0.03204 |
| 1553723_at | GPR97 | G protein-coupled receptor 97 | -1.00118 | 0.041318 |
| 1553861_at | TCP11L2 | t-complex 11 (mouse)-like 2 | 1.137328 | 0.009487 |
| 1554508_at | PIK3AP1 | phosphoinositide-3-kinase adaptor protein 1 | -1.05857 | 0.014124 |
| 1555068_at | WNK1 | WNK lysine deficient protein kinase 1 | 1.507248 | 0.002245 |
| 1555638_a_at | SAMSN1 | SAM domain, SH3 domain and nuclear localization signals 1 | -1.26512 | 0.000452 |
| 1556185_a_at | STEAP4 | STEAP family member 4 | -1.38583 | 0.004856 |
| 1559051_s_at | MB21D1 | Mab-21 domain containing 1 | -1.48246 | 0.000364 |
| 1559573_at | LOC100506229 | hypothetical LOC100506229 | -2.02288 | 0.03258 |
| 1560679_at | LOC100506328 | hypothetical LOC100506328 | -1.56791 | 0.013894 |
| 1568780_at | LOC497257 | hypothetical LOC497257 | -1.05313 | 0.017759 |
| 201041_s_at | DUSP1 | dual specificity phosphatase 1 | -1.16822 | 0.003644 |
| 201052_s_at | PSMF1 | proteasome (prosome, macropain) inhibitor subunit 1 (PI31) | 1.021332 | 0.004112 |
| 201053_s_at | PSMF1 | proteasome (prosome, macropain) inhibitor subunit 1 (PI31) | 1.088499 | 0.001398 |
| 201122_x_at | EIF5A | eukaryotic translation initiation factor 5A | 1.045381 | 0.007668 |
| 201123_s_at | EIF5A | eukaryotic translation initiation factor 5A | 2.023113 | 0.002804 |
| 201249_at | SLC2A1 | solute carrier family 2 (facilitated glucose transporter), member 1 | 1.226607 | 0.027045 |
| 201250_s_at | SLC2A1 | solute carrier family 2 (facilitated glucose transporter), member 1 | 1.381069 | 0.040169 |
| 201537_s_at | DUSP3 | dual specificity phosphatase 3 | -1.02414 | 0.004364 |
| 201940_at | CPD | carboxypeptidase D | -1.12948 | 0.022789 |
| 201942_s_at | CPD | carboxypeptidase D | -1.33144 | 0.007384 |
| 201943_s_at | CPD | carboxypeptidase D | -1.10191 | 0.036253 |
| 202430_s_at | PLSCR1 | phospholipid scramblase 1 | -1.03577 | 0.012683 |
| 202436_s_at | CYP1B1 | cytochrome P450, family 1, subfamily B, polypeptide 1 | -1.23657 | 0.018207 |
| 202437_s_at | CYP1B1 | cytochrome P450, family 1, subfamily B, polypeptide 1 | -1.66659 | 0.005696 |
| 202441_at | ERLIN1 | ER lipid raft associated 1 | -1.05239 | 0.041369 |
| 202444_s_at | ERLIN1 | ER lipid raft associated 1 | -1.06249 | 0.019336 |
| 202708_s_at | HIST2H2BE | histone cluster 2, H2be | -1.05482 | 0.048115 |
| 202922_at | GCLC | glutamate-cysteine ligase, catalytic subunit | 1.287958 | 0.007823 |
| 202953_at | C1QB | complement component 1, q subcomponent, B chain | -1.64092 | 0.031419 |
| 202974_at | MPP1 | membrane protein, palmitoylated 1, 55kDa | 1.095314 | 0.036845 |
| 203116_s_at | FECH | ferrochelatase | 1.547674 | 0.003416 |
| 203502_at | BPGM | 2,3-bisphosphoglycerate mutase | 1.360559 | 0.003343 |
| 203645_s_at | CD163 | CD163 molecule | -2.22533 | 0.008908 |
| 203973_s_at | CEBPD | CCAAT/enhancer binding protein (C/EBP), delta | -1.0217 | 0.01836 |
| 204187_at | GMPR | guanosine monophosphate reductase | 1.143445 | 0.046489 |
| 204560_at | FKBP5 | FK506 binding protein 5 | -1.06493 | 0.019455 |
| 204720_s_at | DNAJC6 | DnaJ (Hsp40) homolog, subfamily C, member 6 | 1.592844 | 0.025488 |
| 204860_s_at | NAIP | NLR family, apoptosis inhibitory protein | -1.45983 | 0.020844 |
| 204861_s_at | NAIP | NLR family, apoptosis inhibitory protein | -1.11548 | 0.021331 |
| 204924_at | TLR2 | toll-like receptor 2 | -1.05081 | 0.015466 |
| 205006_s_at | NMT2 | N-myristoyltransferase 2 | 1.037399 | 0.027581 |
| 205389_s_at | ANK1 | ankyrin 1, erythrocytic | 1.280485 | 0.049183 |
| 205390_s_at | ANK1 | ankyrin 1, erythrocytic | 1.554131 | 0.012932 |
| 205495_s_at | GNLY | granulysin | -1.24637 | 0.002665 |
| 205592_at | SLC4A1 | solute carrier family 4, anion exchanger, member 1 (erythrocyte membrane protein band 3, Diego blood group) | 1.258913 | 0.026053 |
| 205786_s_at | ITGAM (includes EG:16409) | integrin, alpha M (complement component 3 receptor 3 subunit) | -1.03851 | 0.003301 |
| 205900_at | KRT1 | keratin 1 | 1.436683 | 0.020963 |
| 205950_s_at | CA1 | carbonic anhydrase I | 1.725623 | 0.007122 |
| 206464_at | BMX | BMX non-receptor tyrosine kinase | -1.13424 | 0.045286 |
| 206522_at | MGAM | maltase-glucoamylase (alpha-glucosidase) | -1.01163 | 0.024929 |
| 206618_at | IL18R1 | interleukin 18 receptor 1 | -1.64143 | 0.014989 |
| 206698_at | XK | X-linked Kx blood group (McLeod syndrome) | 1.014307 | 0.011568 |
| 206974_at | CXCR6 | chemokine (C-X-C motif) receptor 6 | -1.01897 | 0.014958 |
| 206978_at | CCR2 | chemokine (C-C motif) receptor 2 | -1.34674 | 0.002939 |
| 206991_s_at | CCR5 | chemokine (C-C motif) receptor 5 | -1.0788 | 0.014547 |
| 207008_at | CXCR2 | chemokine (C-X-C motif) receptor 2 | -1.09498 | 0.009814 |
| 207275_s_at | ACSL1 | acyl-CoA synthetase long-chain family member 1 | -1.33457 | 0.017836 |
| 207338_s_at | ZNF200 | zinc finger protein 200 | -1.02999 | 0.029458 |
| 207387_s_at | GK | glycerol kinase | -1.27151 | 0.02265 |
| 207459_x_at | GYPB | glycophorin B (MNS blood group) | 1.494415 | 0.024318 |
| 207791_s_at | RAB1A | RAB1A, member RAS oncogene family | -1.00301 | 0.005529 |
| 207794_at | CCR2 | chemokine (C-C motif) receptor 2 | -1.04133 | 0.002106 |
| 208352_x_at | ANK1 | ankyrin 1, erythrocytic | 1.023101 | 0.029092 |
| 208451_s_at | C4B | complement component 4B (Chido blood group) | 1.765881 | 0.01218 |
| 208488_s_at | CR1 | complement component (3b/4b) receptor 1 (Knops blood group) | -1.03887 | 0.026963 |
| 209189_at | FOS | FBJ murine osteosarcoma viral oncogene homolog | -1.06279 | 0.003695 |
| 209273_s_at | ISCA1 | iron-sulfur cluster assembly 1 homolog (S. cerevisiae) | 1.094893 | 0.045562 |
| 209392_at | ENPP2 | ectonucleotide pyrophosphatase/phosphodiesterase 2 | -1.1497 | 0.015576 |
| 209480_at | HLA-DQB1 | major histocompatibility complex, class II, DQ beta 1 | -2.78201 | 0.03157 |
| 209735_at | ABCG2 | ATP-binding cassette, sub-family G (WHITE), member 2 | 1.419663 | 0.03259 |
| 209845_at | MKRN1 | makorin ring finger protein 1 | 1.078957 | 0.000925 |
| 209960_at | HGF | hepatocyte growth factor (hepapoietin A; scatter factor) | -1.39847 | 0.006502 |
| 210112_at | HPS1 (includes EG:114638) | Hermansky-Pudlak syndrome 1 | 1.146288 | 0.018369 |
| 210119_at | KCNJ15 | potassium inwardly-rectifying channel, subfamily J, member 15 | -1.13616 | 0.033481 |
| 210151_s_at | DYRK3 | dual-specificity tyrosine-(Y)-phosphorylation regulated kinase 3 | 1.025378 | 0.049406 |
| 210164_at | GZMB | granzyme B (granzyme 2, cytotoxic T-lymphocyte-associated serine esterase 1) | -1.20657 | 0.005892 |
| 210504_at | KLF1 | Kruppel-like factor 1 (erythroid) | 1.687688 | 0.017852 |
| 210746_s_at | EPB42 | erythrocyte membrane protein band 4.2 | 1.500856 | 0.017652 |
| 210772_at | FPR2 | formyl peptide receptor 2 | -1.12125 | 0.001556 |
| 210773_s_at | FPR2 | formyl peptide receptor 2 | -1.2273 | 0.002234 |
| 211372_s_at | IL1R2 | interleukin 1 receptor, type II | -2.09981 | 0.032008 |
| 211571_s_at | VCAN | versican | -1.15477 | 0.01001 |
| 211574_s_at | CD46 | CD46 molecule, complement regulatory protein | -1.13302 | 0.047014 |
| 211820_x_at | GYPA | glycophorin A (MNS blood group) | 1.303591 | 0.048908 |
| 211821_x_at | GYPA | glycophorin A (MNS blood group) | 1.532413 | 0.035832 |
| 211990_at | HLA-DPA1 | major histocompatibility complex, class II, DP alpha 1 | -1.37043 | 0.002396 |
| 212148_at | PBX1 | pre-B-cell leukemia homeobox 1 | 1.528451 | 0.030925 |
| 212192_at | KCTD12 | potassium channel tetramerisation domain containing 12 | -1.10503 | 0.01036 |
| 212602_at | WDFY3 | WD repeat and FYVE domain containing 3 | -1.0177 | 0.025199 |
| 212777_at | SOS1 | son of sevenless homolog 1 (Drosophila) | -1.56428 | 0.002406 |
| 212820_at | DMXL2 | Dmx-like 2 | -1.10462 | 0.021551 |
| 213418_at | HSPA6 | heat shock 70kDa protein 6 (HSP70B') | -1.32745 | 0.003983 |
| 213608_s_at | SRRD | SRR1 domain containing | 1.110322 | 0.003904 |
| 213724_s_at | PDK2 | pyruvate dehydrogenase kinase, isozyme 2 | 1.235411 | 0.025701 |
| 213757_at | EIF5A | eukaryotic translation initiation factor 5A | -1.13203 | 0.04263 |
| 213817_at | IRAK3 | interleukin-1 receptor-associated kinase 3 | -1.44559 | 0.003521 |
| 213934_s_at | ZNF23 | zinc finger protein 23 (KOX 16) | 1.235993 | 0.022353 |
| 214407_x_at | GYPB | glycophorin B (MNS blood group) | 1.6232 | 0.025513 |
| 214428_x_at | C4B | complement component 4B (Chido blood group) | 1.663075 | 0.009039 |
| 214433_s_at | SELENBP1 | selenium binding protein 1 | 1.292835 | 0.024764 |
| 214472_at | HIST1H3A | histone cluster 1, H3a | -1.35661 | 0.01691 |
| 214511_x_at | FCGR1B | Fc fragment of IgG, high affinity Ib, receptor (CD64) | -1.1051 | 0.038902 |
| 214590_s_at | UBE2D1 | ubiquitin-conjugating enzyme E2D 1 | -1.11855 | 0.023272 |
| 214706_at | ZNF200 | zinc finger protein 200 | -1.0834 | 0.007464 |
| 215049_x_at | CD163 | CD163 molecule | -2.30284 | 0.00677 |
| 215242_at | PIGC | phosphatidylinositol glycan anchor biosynthesis, class C | 1.38401 | 0.025327 |
| 215646_s_at | VCAN | versican | -1.16842 | 0.006501 |
| 215761_at | DMXL2 | Dmx-like 2 | -1.00802 | 0.020508 |
| 215990_s_at | BCL6 | B-cell CLL/lymphoma 6 | -1.24455 | 0.001712 |
| 216233_at | CD163 | CD163 molecule | -1.18121 | 0.017939 |
| 216317_x_at | RHCE/RHD | Rh blood group, D antigen | 1.155257 | 0.02433 |
| 216833_x_at | GYPB | glycophorin B (MNS blood group) | 1.529166 | 0.024352 |
| 216899_s_at | SKAP2 | src kinase associated phosphoprotein 2 | -1.02655 | 0.024062 |
| 217104_at | ST20 | suppressor of tumorigenicity 20 | -1.22745 | 0.03595 |
| 217437_s_at | TACC1 | transforming, acidic coiled-coil containing protein 1 | -1.04633 | 0.043888 |
| 218116_at | C9orf78 | chromosome 9 open reading frame 78 | 1.131912 | 0.009344 |
| 218141_at | UBE2O | ubiquitin-conjugating enzyme E2O | 1.092201 | 0.018805 |
| 218232_at | C1QA | complement component 1, q subcomponent, A chain | -1.48377 | 0.015729 |
| 218454_at | PLBD1 | phospholipase B domain containing 1 | -1.01617 | 0.000647 |
| 218864_at | TNS1 | tensin 1 | 1.40982 | 0.021017 |
| 218918_at | MAN1C1 | mannosidase, alpha, class 1C, member 1 | 1.022559 | 0.026804 |
| 219386_s_at | SLAMF8 | SLAM family member 8 | -1.03625 | 0.014004 |
| 219546_at | BMP2K | BMP2 inducible kinase | 1.033265 | 0.021759 |
| 219666_at | MS4A6A | membrane-spanning 4-domains, subfamily A, member 6A | -1.03153 | 0.004294 |
| 219672_at | AHSP | alpha hemoglobin stabilizing protein | 1.133232 | 0.018751 |
| 219890_at | CLEC5A | C-type lectin domain family 5, member A | -1.41646 | 0.032089 |
| 219938_s_at | PSTPIP2 | proline-serine-threonine phosphatase interacting protein 2 | -1.26104 | 0.010536 |
| 219975_x_at | OLAH | oleoyl-ACP hydrolase | -1.4701 | 0.044801 |
| 220034_at | IRAK3 | interleukin-1 receptor-associated kinase 3 | -1.06465 | 0.013564 |
| 220173_at | C14orf45 | chromosome 14 open reading frame 45 | 1.49005 | 0.003199 |
| 220330_s_at | SAMSN1 | SAM domain, SH3 domain and nuclear localization signals 1 | -1.18478 | 0.002472 |
| 220751_s_at | C5orf4 | chromosome 5 open reading frame 4 | 1.534989 | 0.006816 |
| 220832_at | TLR8 | toll-like receptor 8 | -1.13198 | 0.003801 |
| 221237_s_at | OSBP2 | oxysterol binding protein 2 | 1.210608 | 0.041056 |
| 221478_at | BNIP3L | BCL2/adenovirus E1B 19kDa interacting protein 3-like | 1.068535 | 0.007646 |
| 221627_at | TRIM10 | tripartite motif containing 10 | 1.230021 | 0.011252 |
| 221747_at | TNS1 | tensin 1 | 1.495251 | 0.027967 |
| 221748_s_at | TNS1 | tensin 1 | 1.586389 | 0.023444 |
| 221824_s_at | MARCH8 | membrane-associated ring finger (C3HC4) 8 | 1.026101 | 0.013806 |
| 221932_s_at | GLRX5 | glutaredoxin 5 | 1.016878 | 0.028989 |
| 222496_s_at | RBM47 | RNA binding motif protein 47 | -1.02925 | 0.026022 |
| 222687_s_at | ACER3 | alkaline ceramidase 3 | -1.02925 | 0.012769 |
| 222693_at | FNDC3B | fibronectin type III domain containing 3B | -1.02964 | 0.006019 |
| 222721_at | CNIH4 | cornichon homolog 4 (Drosophila) | -1.08283 | 0.01642 |
| 222895_s_at | BCL11B | B-cell CLL/lymphoma 11B (zinc finger protein) | 1.347469 | 0.037271 |
| 222945_x_at | OLAH | oleoyl-ACP hydrolase | -1.15684 | 0.015626 |
| 223124_s_at | PITHD1 | PITH (C-terminal proteasome-interacting domain of thioredoxin-like) domain containing 1 | 1.433289 | 0.008575 |
| 223204_at | FAM198B | family with sequence similarity 198, member B | -1.04469 | 0.013971 |
| 223432_at | OSBP2 | oxysterol binding protein 2 | 1.648538 | 0.008885 |
| 223597_at | ITLN1 | intelectin 1 (galactofuranose binding) | 1.5827 | 0.041377 |
| 223669_at | HEMGN | hemogen | 1.628387 | 0.034946 |
| 223670_s_at | HEMGN | hemogen | 1.782966 | 0.00141 |
| 223952_x_at | DHRS9 | dehydrogenase/reductase (SDR family) member 9 | -1.03108 | 0.016567 |
| 224009_x_at | DHRS9 | dehydrogenase/reductase (SDR family) member 9 | -1.12083 | 0.009459 |
| 224314_s_at | EGLN1 | egl nine homolog 1 (C. elegans) | -1.03633 | 0.004375 |
| 224341_x_at | TLR4 | toll-like receptor 4 | -1.18434 | 0.018445 |
| 224891_at | FOXO3 | forkhead box O3 | 1.034798 | 0.023177 |
| 224898_at | WDR26 | WD repeat domain 26 | 1.181848 | 0.045529 |
| 224905_at | WDR26 | WD repeat domain 26 | 1.187785 | 0.017186 |
| 225051_at | EPB41 | erythrocyte membrane protein band 4.1 (elliptocytosis 1, RH-linked) | 1.062133 | 0.005264 |
| 225056_at | SIPA1L2 | signal-induced proliferation-associated 1 like 2 | -1.40641 | 0.015878 |
| 225353_s_at | C1QC | complement component 1, q subcomponent, C chain | -1.39915 | 0.020331 |
| 225387_at | TSPAN5 | tetraspanin 5 | 1.275052 | 0.00898 |
| 225878_at | KIF1B | kinesin family member 1B | -1.03193 | 0.006232 |
| 226416_at | ERI1 (includes EG:361159) | exoribonuclease 1 | -1.16874 | 0.025507 |
| 226825_s_at | TMEM165 | transmembrane protein 165 | -1.05597 | 0.002719 |
| 227250_at | KREMEN1 | kringle containing transmembrane protein 1 | -1.00468 | 0.001465 |
| 227309_at | YOD1 | YOD1 OTU deubiquinating enzyme 1 homolog (S. cerevisiae) | 1.04528 | 0.040356 |
| 227769_at | GPR27 | G protein-coupled receptor 27 | -1.32022 | 0.017697 |
| 228188_at | FOSL2 | FOS-like antigen 2 | -1.0208 | 0.003894 |
| 228220_at | FCHO2 | FCH domain only 2 | -1.18086 | 0.036266 |
| 228758_at | BCL6 | B-cell CLL/lymphoma 6 | -1.03138 | 0.040476 |
| 228770_at | GPR146 | G protein-coupled receptor 146 | 1.196172 | 0.019806 |
| 228996_at | RC3H1 | ring finger and CCCH-type domains 1 | -1.02059 | 0.048389 |
| 229005_at | MCTP2 | multiple C2 domains, transmembrane 2 | -1.12717 | 0.011844 |
| 229228_at | CREB5 | cAMP responsive element binding protein 5 | -1.16456 | 0.020361 |
| 231874_at | FAM126B | family with sequence similarity 126, member B | -1.05708 | 0.032456 |
| 231933_at | MARCH8 | membrane-associated ring finger (C3HC4) 8 | 1.066229 | 0.017985 |
| 231982_at | C19orf77 | chromosome 19 open reading frame 77 | 1.085092 | 0.007797 |
| 231997_at | TBCEL | tubulin folding cofactor E-like | 1.006768 | 0.02002 |
| 232068_s_at | TLR4 | toll-like receptor 4 | -1.30127 | 0.022209 |
| 233126_s_at | OLAH | oleoyl-ACP hydrolase | -1.31599 | 0.029539 |
| 233587_s_at | SIPA1L2 | signal-induced proliferation-associated 1 like 2 | -1.34934 | 0.026226 |
| 234362_s_at | CTLA4 | cytotoxic T-lymphocyte-associated protein 4 | -1.24035 | 0.041415 |
| 235514_at | ASPRV1 | aspartic peptidase, retroviral-like 1 | -1.1891 | 0.032015 |
| 236081_at | SNCA | synuclein, alpha (non A4 component of amyloid precursor) | 1.440437 | 0.044911 |
| 238439_at | ANKRD22 | ankyrin repeat domain 22 | -1.32664 | 0.046381 |
| 238513_at | PRRG4 | proline rich Gla (G-carboxyglutamic acid) 4 (transmembrane) | -1.39075 | 0.048646 |
| 238858_at | TIFA | TRAF-interacting protein with forkhead-associated domain | -1.10957 | 0.019324 |
| 239206_at | CR1L | complement component (3b/4b) receptor 1-like | 1.631136 | 0.009521 |
| 241817_at | C3orf62 | chromosome 3 open reading frame 62 | -1.03078 | 0.019329 |
| 241881_at | OR2W3 | olfactory receptor, family 2, subfamily W, member 3 | 1.507999 | 0.047705 |
| 37145_at | GNLY | granulysin | -1.0927 | 0.00544 |
| 39729_at | PRDX2 | peroxiredoxin 2 | 1.046544 | 0.01548 |
| 59644_at | BMP2K | BMP2 inducible kinase | 1.08296 | 0.039044 |
