# Supplementary material for: Expression of complement and toll-like receptor pathway genes is associated with malaria severity in Mali: a pilot case control study
Source: Malar J. 2016 Mar 9;15:150. doi: 10.1186/s12936-016-1189-6 (PMC4784286; doi:10.1186/s12936-016-1189-6)
Supplement: Supplementary file 5 — 10.1186/s12936-016-1189-6 Top differentially expressed KEGG pathways between severe cases and uncomplicated controls during convalescence. [file 12936_2016_1189_MOESM5_ESM.docx]

**Table S4 Top differentially expressed KEGG pathways between severe cases and uncomplicated controls during convalescence**

| **Kegg Pathway** | **Size** | **Expected Count** | **Observed Count** | **Odds Ratio** | **p value** |
| --- | --- | --- | --- | --- | --- |
| Staphylococcus aureus infection | 47 | 0 | 5 | 16.401 | <0.001 |
| Asthma | 26 | 0 | 4 | 24.436 | <0.001 |
| Intestinal immune network for IgA production | 41 | 0 | 4 | 14.483 | <0.001 |
| Phagosome | 137 | 1 | 6 | 6.378 | 0.001 |
| Antigen processing and presentation | 67 | 1 | 4 | 8.459 | 0.002 |
| Allograft rejection | 33 | 0 | 3 | 13.044 | 0.002 |
| Graft-versus-host disease | 35 | 0 | 3 | 12.224 | 0.003 |
| Type I diabetes mellitus | 39 | 0 | 3 | 10.856 | 0.004 |
| Autoimmune thyroid disease | 47 | 0 | 3 | 8.867 | 0.006 |
| Chemokine signaling pathway | 165 | 1 | 5 | 4.197 | 0.01 |
| Cytokine-cytokine receptor interaction | 234 | 2 | 6 | 3.587 | 0.011 |
| Mismatch repair | 22 | 0 | 2 | 12.719 | 0.014 |
| Leishmaniasis | 66 | 1 | 3 | 6.168 | 0.016 |
| Viral myocarditis | 66 | 1 | 3 | 6.168 | 0.016 |
| Toxoplasmosis | 121 | 1 | 4 | 4.502 | 0.016 |
| Rheumatoid arthritis | 79 | 1 | 3 | 5.099 | 0.026 |
