# Supplementary material for: Expression of complement and toll-like receptor pathway genes is associated with malaria severity in Mali: a pilot case control study
Source: Malar J. 2016 Mar 9;15:150. doi: 10.1186/s12936-016-1189-6 (PMC4784286; doi:10.1186/s12936-016-1189-6)
Supplement: Supplementary file 6 — 10.1186/s12936-016-1189-6 Top differentially expressed KEGG pathways between severe cases and uncomplicated controls during late convalescence. [file 12936_2016_1189_MOESM6_ESM.docx]

**Table S5 Top differentially expressed KEGG pathways between severe cases and uncomplicated controls during late convalescence**

| **KEGG Pathway** | **Size** | **Expected Count** | **Observed Count** | **Odds Ratio** | **p value** |
| --- | --- | --- | --- | --- | --- |
| Hematopoietic cell lineage | 83 | 1 | 7 | 5.982 | <0.001 |
| Toll-like receptor signaling pathway | 97 | 2 | 7 | 5.036 | 0.001 |
| Hepatitis C | 125 | 2 | 7 | 3.817 | 0.004 |
| RIG-I-like receptor signaling pathway | 66 | 1 | 5 | 5.194 | 0.004 |
| Cytosolic DNA-sensing pathway | 48 | 1 | 4 | 5.704 | 0.007 |
| NOD-like receptor signaling pathway | 52 | 1 | 4 | 5.224 | 0.01 |
| Cytokine-cytokine receptor interaction | 234 | 4 | 9 | 2.587 | 0.013 |
| African trypanosomiasis | 31 | 1 | 3 | 6.656 | 0.014 |
| PPAR signaling pathway | 64 | 1 | 4 | 4.168 | 0.02 |
| Type I diabetes mellitus | 39 | 1 | 3 | 5.168 | 0.025 |
| Nicotinate and nicotinamide metabolism | 17 | 0 | 2 | 8.196 | 0.031 |
| Malaria | 48 | 1 | 3 | 4.126 | 0.043 |
| Amyotrophic lateral sclerosis (ALS) | 50 | 1 | 3 | 3.949 | 0.048 |
| Apoptosis | 84 | 1 | 4 | 3.113 | 0.048 |
