# Supplementary material for: Expression of complement and toll-like receptor pathway genes is associated with malaria severity in Mali: a pilot case control study
Source: Malar J. 2016 Mar 9;15:150. doi: 10.1186/s12936-016-1189-6 (PMC4784286; doi:10.1186/s12936-016-1189-6)
Supplement: Supplementary file 7 — 10.1186/s12936-016-1189-6 RT-PCR validation of GeneChip expression data with RT-PCR (probes for CYP1B1, IL18R1 and C1QB failed to consistently amplify, failing the inclusion criteria; therefore they were not included in the final validation analysis). [file 12936_2016_1189_MOESM7_ESM.docx]

**Table S6 RT-PCR validation of GeneChip expression data with RT-PCR (probes for CYP1B1, IL18R1 and C1QB failed to consistently amplify, failing the inclusion criteria; therefore they were not included in the final validation analysis)**

| **Gene** | **Gene Chip Expression** | **RT-PCR Expression** |
| --- | --- | --- |
| CD163 | 0.38 | 0.73 |
| NAIP | 0.32 | 0.49 |
| TLR2 | 0.63 | 0.79 |
| CLEC4D | 0.38 | 0.52 |
| CCR2 | 0.34 | 0.34 |
